# Block Sequence Effects on the Self-Assembly Behaviors of Polypeptide-Based Penta-Block Copolymer Hydrogels

**DOI:** 10.1021/acsami.3c18954

**Published:** 2024-01-30

**Authors:** Ke-Hsin Wang, Chung-Hao Liu, Dun-Heng Tan, Mu-Ping Nieh, Wei-Fang Su

**Affiliations:** † Department of Materials Science and Engineering, National Taiwan University, No. 1, Sec. 4, Roosevelt Road, Taipei 10617, Taiwan; ‡ Polymer program, Institute of Materials Science, University of Connecticut, 25 King Hill Road, Unit 3136, Storrs, Connecticut 06269-3136, United States; § Department of Chemical and Biomolecular Engineering, University of Connecticut, Storrs, Connecticut 06269, United States; ∥ Department of Materials Engineering, Ming-Chi University of Technology, 84 Gungjuan Rd., Taishan Dist, New Taipei City 243303, Taiwan

**Keywords:** hydrogel, block copolymer, self-assembly, micelle, peptide, small-angle X-ray scattering

## Abstract

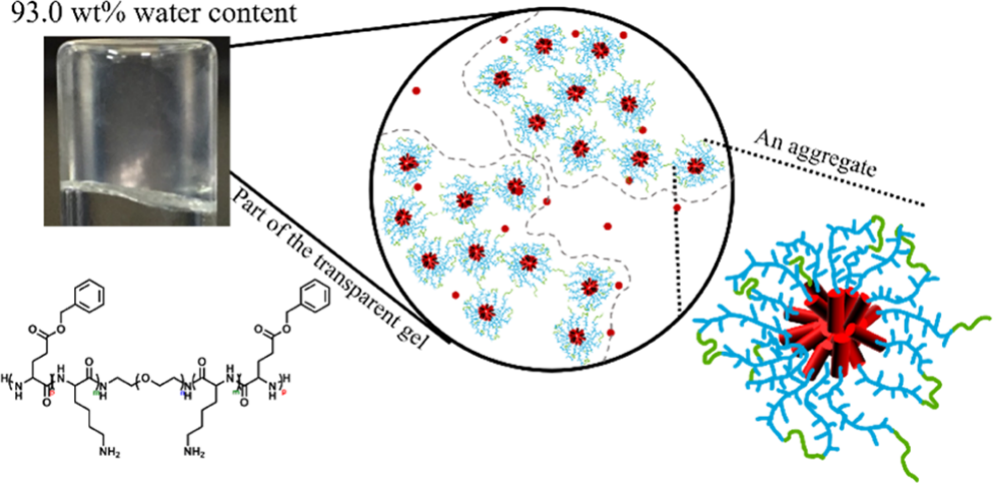

Peptide-based hydrogels have great potential for applications in tissue engineering, drug delivery, and so on. We systematically synthesize, characterize, and investigate the self-assembly behaviors of a series of polypeptide-based penta-block copolymers by varying block sequences and lengths. The copolymers contain hydrophobic blocks of poly(γ-benzyl-l-glutamate) (PBG, B_*x*_) and two kinds of hydrophilic blocks, poly(l-lysine) (PLL, K_*y*_) and poly(ethylene glycol) (PEG, EG_34_), where *x* and *y* are the number of repeating units of each block, where PBG and PLL blocks have unique functions for nerve regeneration and cell adhesion. It shows that a sufficient length of the middle hydrophilic segment capped with hydrophobic end PBG blocks is required. They first self-assemble into flower-like micelles and sequentially form transparent hydrogels (as low as 2.3 wt %) with increased polymer concentration. The hydrogels contain a microscale porous structure, a desired property for tissue engineering to facilitate the access of nutrient flow for cell growth and drug delivery systems with high efficiency of drug storage. We hypothesize that the structure of B_*x*_-K_*y*_-EG_34_-K_*y*_-B_*x*_ agglomerates is beyond micron size (transparent), while that of K_*y*_-B_*x*_-EG_34_-B_*x*_-K_*y*_ is on the submicron scale (opaque). We establish a working strategy to synthesize a polypeptide-based block copolymer with a wide window of sol–gel transition. The study offers insight into rational polypeptide hydrogel design with specific morphology, exploring the novel materials as potential candidates for neural tissue engineering.

## Introduction

Polypeptide-based amphiphilic block copolymers are promising hydrogel biomaterials attributed to their highly versatile self-assembly structure, high biocompatibility, and high biodegradability. Moreover, the balanced forces in materials such as abundant hydrogen bonds and electrostatic forces can determine their physical structures by slight variations in composition, concentration in solution, or environmental conditions.^1−4^ Their dynamic and versatile architectures have attractive applications in controlled drug delivery systems^5,6^ or tissue engineering scaffolds^6,7^ via environmentally stimulated phase transitions. To design these types of biomaterials, researchers tend to incorporate more than two kinds of polypeptides and biocompatible synthetic polymers to tune the desired properties for specific application needs.^5,8−11^ For instance, Rodriguez et al. utilized dual hydrophilic segments to achieve a balance between cellular uptake ability and toxicity in order to optimize the polymeric vesicle properties.^12^ Lin et al. combined oligo(alanine), oligo(lysine), and poly(ethylene glycol)-poly(propylene oxide)-poly(ethylene glycol) (PLX) to develop a thermal responsive self-assembly scaffold for sustained drug release.^6^ As biomaterials become more multifunctional, designing multiblock copolymers to fine-tune cell viability and self-assembly architecture has become a trend in developing new materials, a goal that acts as a stepping stone for proceeding into sequence-controlled block copolymers.^13,14^

Biomaterial functionalities highly depend on their self-assembly structures; the interconnecting network is ideal for extracellular matrix (ECM) mimicking scaffolds, while vesicles and micelles would be more preferred in a drug-delivery system.^15,16^ However, it is challenging to predict and design desired structures, as self-assembly behaviors are versatile and sensitive to environmental conditions. Currently, researchers mostly rely on the results of experiments to understand the trend of structural changes and finally conclude the understanding with a model that appropriately describes the self-assembly mechanism. For instance, some work demonstrated β-sheet strengthening at an elevated temperature, making it applicable to thermosensitive hydrogels.^17,18^ Gelation also occurs in polymers containing polyelectrolytes. The polymer can be used as a tissue engineering scaffold, and the electrostatic force is accounted for the solid fibril architecture.^19,20^ Hydrophobic interaction-induced aggregation can form gelation through micelle formation.^21−23^ Other mechanisms include the coiled-coil association^24^ and the polyion complexation.^25^ In many cases, the development of a novel material relies on extensive trial-and-error experiments to obtain a desired architecture due to the difficulties in predicting self-assembly behaviors.

Accurate prediction of the self-assembly architecture of the multiblock copolymer in solution is relatively rare mostly because the multiblock copolymer systems are extremely complicated.^26^ Fundamental understandings of the relationship between chemical compositions, physical structural arrangements (e.g., block sequence and the length of each block), and hydrophobicity of the copolymers can guide the rational design of their self-assemblies into the desired and controllable morphology. Research has shown that an increase in the number of blocks generally leads to enhanced mechanical properties in the formation of hydrogels.^1,25^ It is known that the arrangement of blocks affects the secondary structure,^27^ water solubility,^27,28^ and drug release efficiency.^29^ It is therefore anticipated that the block sequence can tune the gelation concentration and sol–gel transition. While little research attention is paid to the block arrangement within the polymer chain, this report hopes to provide the missing piece in the puzzle of the self-assembly multiblock polymer structure in aqueous solution. We developed a penta-block copolymer system to examine the impact of the block sequence on morphology and architecture, from which we can design a block copolymer hydrogel with a wider gelation window for property tuning.

Aiming to design targeting biomaterials for neural tissue engineering, we chose poly(γ-benzyl-l-glutamate) (PBG) as the hydrophobic block for its outstanding effect on stimulating the growth of neural cells. In our previous study, we successfully fabricated electrospun PBG into a biomimetic scaffold for nerve regeneration.^30−34^ Through its low immune response and efficient guidance of neurite growth, PBG exhibited its capability for optic^31,34^ and corneal nerve regeneration.^32^ The block copolymers of PBG and poly(l-lysine) (PLL) can improve the biocompatibility of the PBG due to the enhanced adhesion from polylysine.^33,35^ The strong hydrophobicity of PBG can form hydrogels with relatively low water content (<50 wt %) as being block copolymerized with poly(ethylene glycol) (PEG)^36^ or PLX.^37^ Here, we utilized dual hydrophilic polymer segments, PEG and PLL to enhance water solubility and foster cell attachment.^38^ While PLL might increase cellular toxicity due to its cationic nature,^39^ PEG, which is also hydrophilic, is applied to tune the ratio of hydrophilic and hydrophobic independent of ionic concentration. Utilizing the aforementioned segments as the building blocks, we investigate the block sequence effect of such polymers through nanoscale structural analysis to design novel biomaterials for potential application in neural tissue engineering.

## Results and Discussion

### Synthesis and Characterization of Block Copolymers

**Figure 1:**
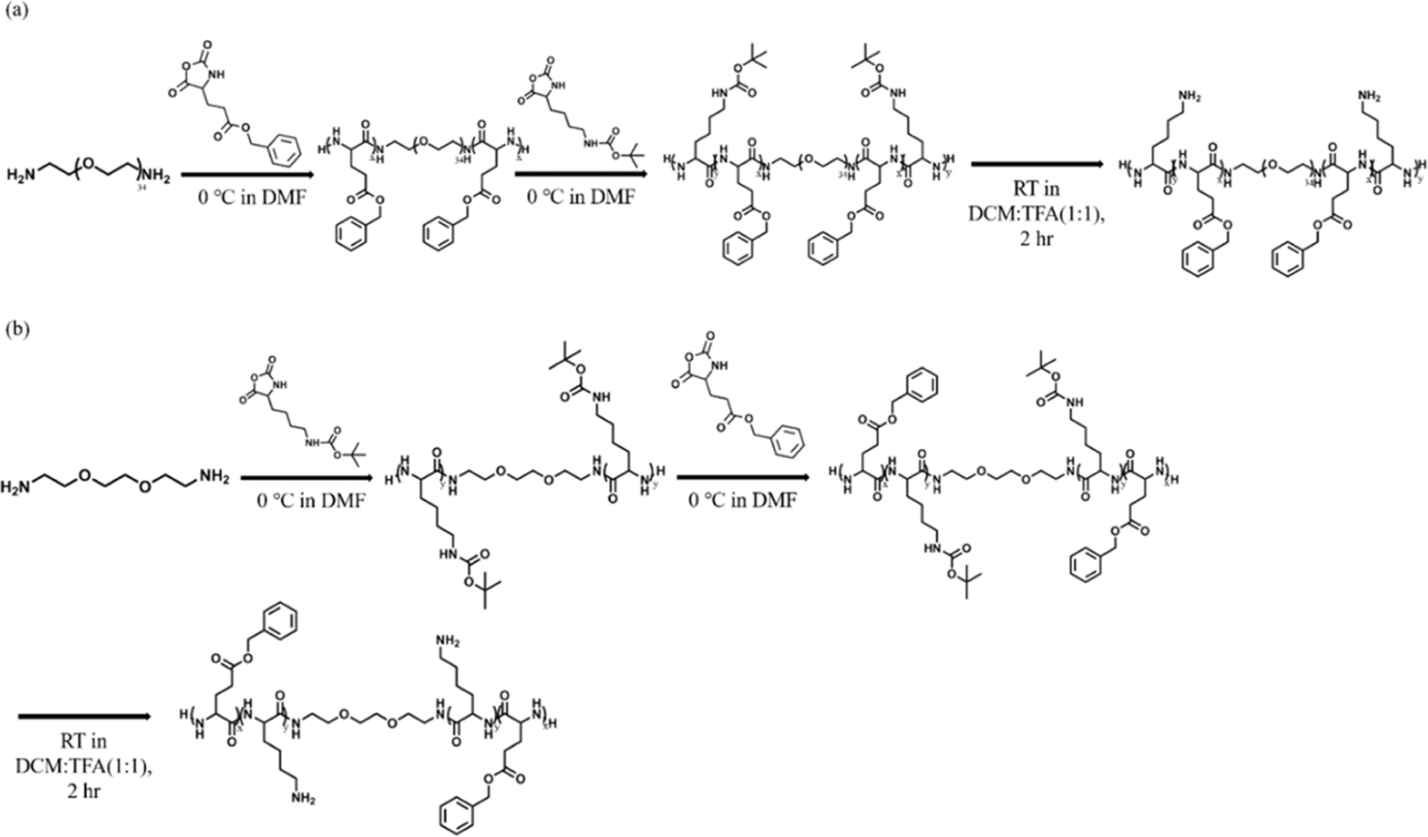
Synthetic routes to (a) K_*y*_-B_*x*_-EG_34_-B_*x*_-K_*y*_ and (b) B_*x*_-K_*y*_-EG_2_-K_*y*_-B_*x*_ copolymers. Well-defined penta-block copolymers are synthesized by sequential addition of two monomers. Random copolymers, (B_*x*_-*r*-K_*y*_)-EG_34_-(B_*x*_-*r*-K_*y*_), are synthesized by reacting two monomers at once.

The polypeptide-containing block copolymers were synthesized by sequential ring-opening polymerization under controlled reaction conditions (Figures 1 and S1). Details of the synthetic procedures of copolymers are described in the Experimental Methods section. The chemical structures of the macroinitiator, monomers, and resultant polymers were confirmed by ^1^H NMR (Figures S2 and S3) and Fourier transform infrared spectroscopy (FT-IR) (Figure S4). The molecular weight distribution of the polymers was kept narrow in order to foster their self-assembly and self-healing properties when the hydrogel is formed.^22,25^

**Figure 2:**
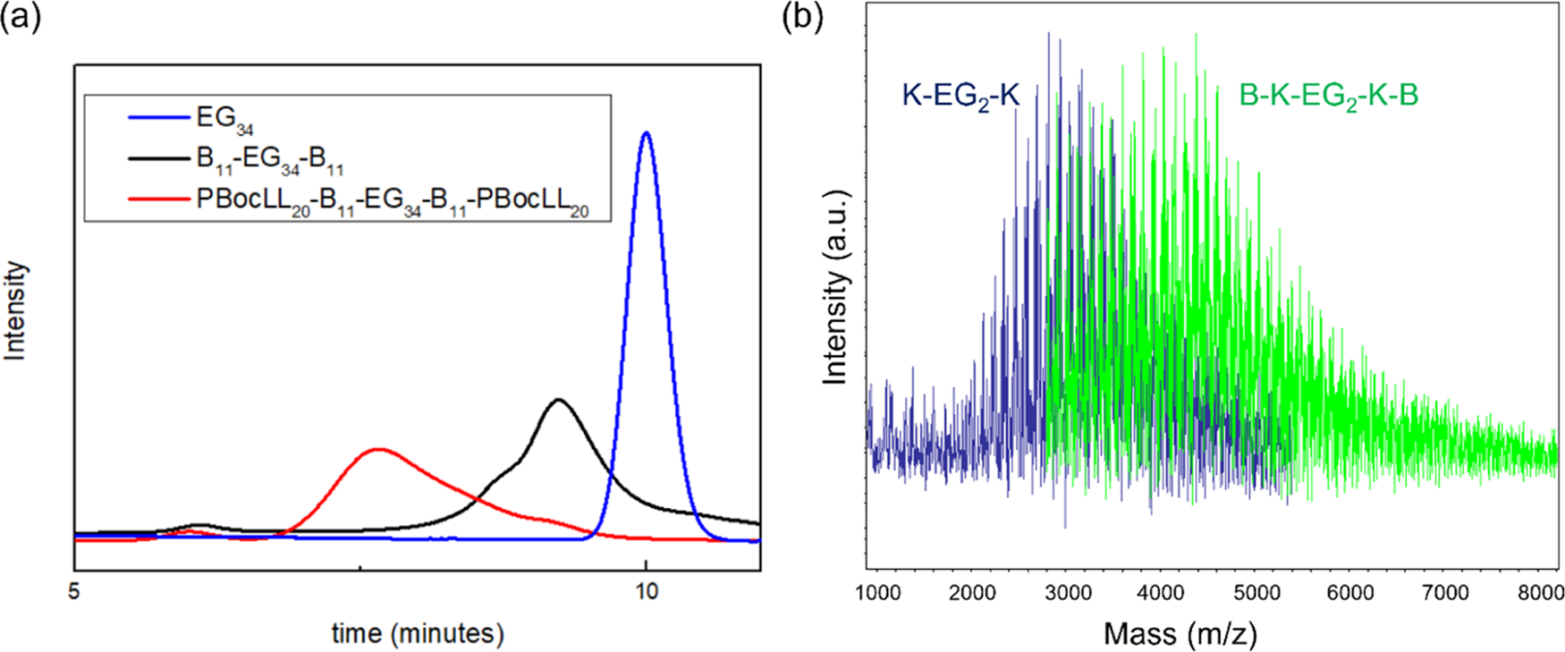
Confirmation of sequential polymerization. (a) GPC trace of PBocLL_*y*_-B_*x*_-EG_34_-B_*x*_-PBocLL_*y*_ in comparison with the product before the first and second monomer addition. PBocLL: poly(N^ε^-*tert*-butyloxycarbonyl-l-lysine). Note that the minor peak observed at a retention time of 6 min is hypothesized to represent polymer agglomeration that occurred within the solution during the GPC measurement.^40−42^ (b) MALDI-ToF-MS spectra of B_13_–K_20_-EG_2_-K_20_-B_13_ (green) and K_20_-EG_2_-K_20_ (blue) with the CHCA matrix. The spectra demonstrate a shift after the second monomer is added.

**Figure 3:**
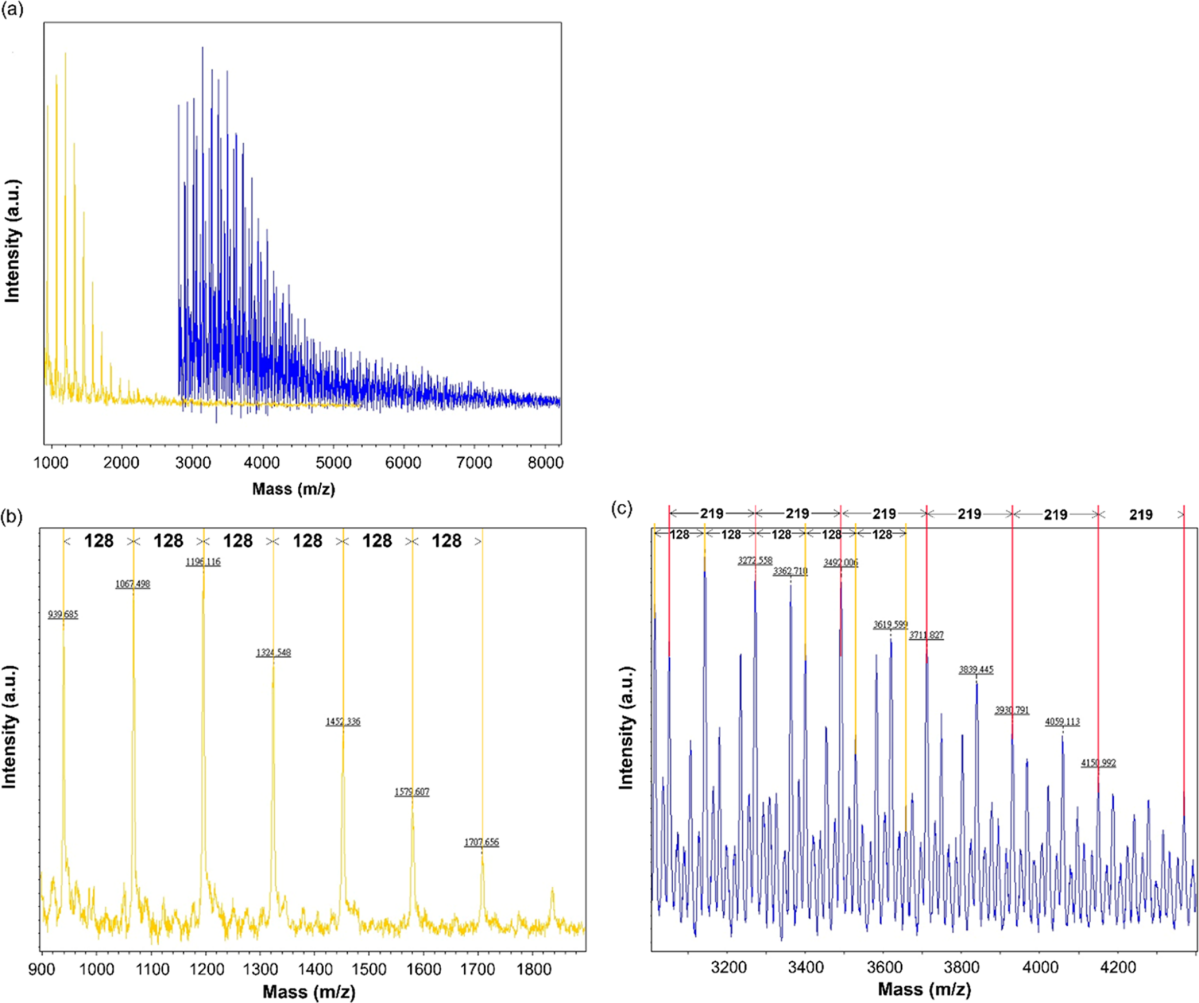
MALDI-ToF-MS spectra of copolymers B_13_–K_20_-EG_2_-K_20_-B_13_ (blue) and K_20_-EG_2_-K_20_ copolymers (yellow) with the SA matrix. (a) The distribution of the two polymers. (b) Spacing of 128 in the spectra of K_20_-EG_2_-K_20_ indicates that polymers have r.u. with a molecular weight of 128 Da, which is exactly the molecular weight of one r.u. of PLL. (c) The spacings of 128 and 219 in the spectra of B_13_–K_20_-EG_2_-K_20_-B_13_ refer to the molecular weight of one r.u. of PLL and PBG, respectively.

Three types of copolymers were synthesized: (B_*x*_-*r*-K_*y*_)-EG_34_-(B_*x*_-*r*-K_*y*_), K_*y*_-B_*x*_-EG_34_-B_*x*_-K_*y*_, and B_*x*_-K_*y*_-EG_34_-K_*y*_-B_*x*_, where B, K, and EG are γ-benzyl-l-glutamate, l-lysine, and ethylene glycol repeat units (r.u.), respectively, and B_*x*_-*r*-K_*y*_ indicates that the random block is made of B_*x*_ and K_*y*_. The block arrangement is confirmed by gel permeation chromatography (GPC) and matrix-assisted laser desorption/ionization time-of-flight mass spectroscopy (MALDI-ToF-MS). The peak position of the GPC trace shifts to a high-molecular-weight end, indicating the success of sequential polymerization in two steps. In the study of MALDI-ToF-MS, α-cyano-4-hydroxycinnamic acid (CHCA) was utilized as the matrix to assist ionization and desorption of copolymer as shown in Figure 2b. When using sinapic acid (SA) instead, a smaller amount of high-molecular-weight polymers was successfully desorbed, but a high-resolution spectrum can be obtained (Figure 3). Only the spacing with *m*/*z* = 128 can be found in the distribution of K_20_-EG_2_-K_20_ (yellow), indicating that the polymer is composed of the PLL repeat units (r.u.). In the distribution of B_13_–K_20_-EG_2_-K_20_-B_13_, two spacings can be identified. They are *m*/*z* = 128 and *m*/*z* = 219, corresponding to the number of lysine r. u. and that of benzyl glutamate r. u., respectively. Both GPC and MALDI-ToF-MS prove that the penta-block arrangement is obtained for the synthesized copolymer.

### Tendency to Gel Formation of Three Different Block Sequences

**Figure 4:**
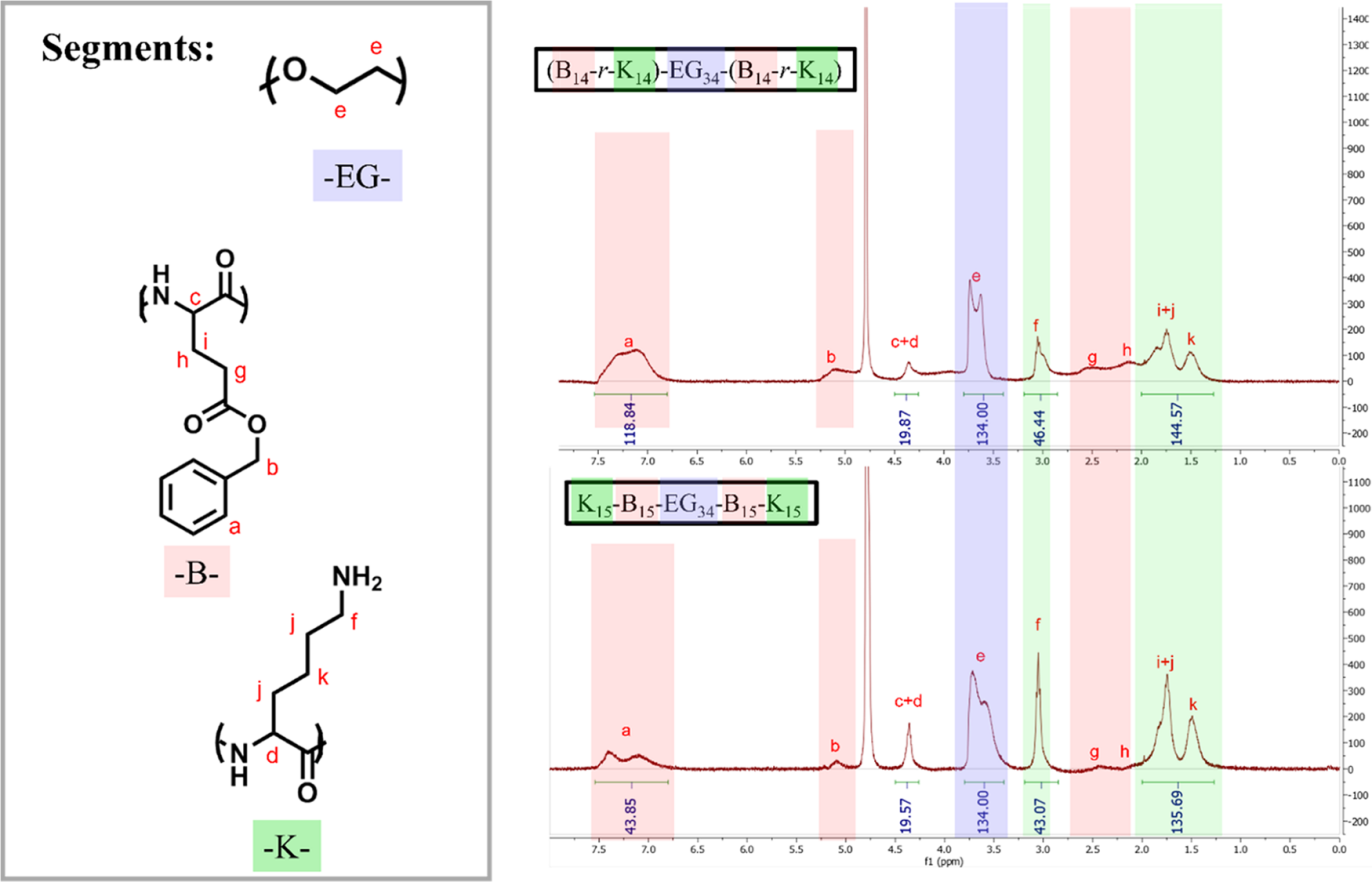
^1^H NMR (400 MHz) of the (B_14_-*r*-K_14_)-EG_34_-(B_14_-*r*-K_14_) copolymer and K_15_–B_15_-EG_34_-B_15_–K_15_ copolymer in D_2_O. Due to aggregation, peaks of PBG and PLL both shrink, as shown in the spectrum of the (B_14_-*r*-K_14_)-EG_34_-(B_14_-*r*-K_14_) copolymer, while the peaks of PBG shrink to a larger extent as shown in the spectrum of the K_15_–B_15_-EG_34_-B_15_–K_15_ copolymer.

Park et al. showed that the sequence of blocks leads to a difference in aggregation shapes, which results in distinctive gelation conditions and thermosensitive properties.^27^ Herein, we demonstrate a dramatic structural change when different block arrangements are applied in block copolymers. Samples with the same monomer compositions but different block arrangements were investigated. The (B_14_-*r*-K_14_)-EG_34_-(B_14_-*r*-K_14_) aqueous solution (1 wt %) is transparent. On the contrary, the copolymer with a well-defined block sequence, K_15_–B_15_-EG_34_-B_15_–K_15_ of the same concentration, appears opaque in aqueous solution, suggesting large-sized aggregates of submicrometer to micrometer range with a distinct refractive index from that of the solvent (i.e., water). Such aggregation can be explained by ^1^H NMR using D_2_O as a solvent (Figure 4). The peaks of polypeptides in the green and red zones in Figure 4 become broadened or diminish when the electrons are shielded differently in aggregated polymer chains, and the reduced area of the peaks reveals a loss of mobility of the corresponding segments.^43^ Peaks of PBG (in the red zone) reduce significantly in the spectrum of K_15_–B_15_-EG_34_-B_15_–K_15_ (lower figure) in comparison with those of (B_14_-*r*-K_14_)-EG_34_-(B_14_-*r*-K_14_) (upper figure), indicating that polymer aggregation takes places around the B_15_ segment in the former case.

The secondary structure of K_15_–B_15_-EG_34_-B_15_–K_15_ is examined through circular dichroism (CD) spectroscopy (Figure S5) using a 0.01 wt % polymer solution in deionized water. CD outcome reveals a predominant α helix secondary structure in K_15_–B_15_-EG_34_-B_15_–K_15_, evidenced by negative peaks at 208 and 222 nm. The adoption of an α helix structure by PBG segments is noted,^41^ likely contributing to this secondary structure. The positive peak of the standard α helix at 190 nm is likely counteracted by the negative peak of the random-coil structure, suggesting the presence of PLL segments. The spectrum of K_15_–B_15_-EG_34_-B_15_–K_15_ appears more red-shifted and distorted than its counterpart, suggesting chain aggregation.^44,45^

The presence of the secondary structure α-helix of the polypeptide has been shown to improve its biocompatibility.^46,47^ Moreover, the secondary structure of a polypeptide can also minimize the antibody response, highlighting its potential in biohybrid drug delivery.^48^

Furthermore, the α-helix polymer-based vehicle outperformed the random-coil vehicle on the intracellular gene-delivery performance, emphasizing the importance of the secondary structure of polypeptides.^49^ In this study, the block copolymer exhibited a stronger secondary α-helix structure than the random copolymer as indicated by the CD spectrum, implying a higher stability in the water solution. The novel block copolymers are expected to show more versatile controllable structures over random copolymers.

**Figure 5:**
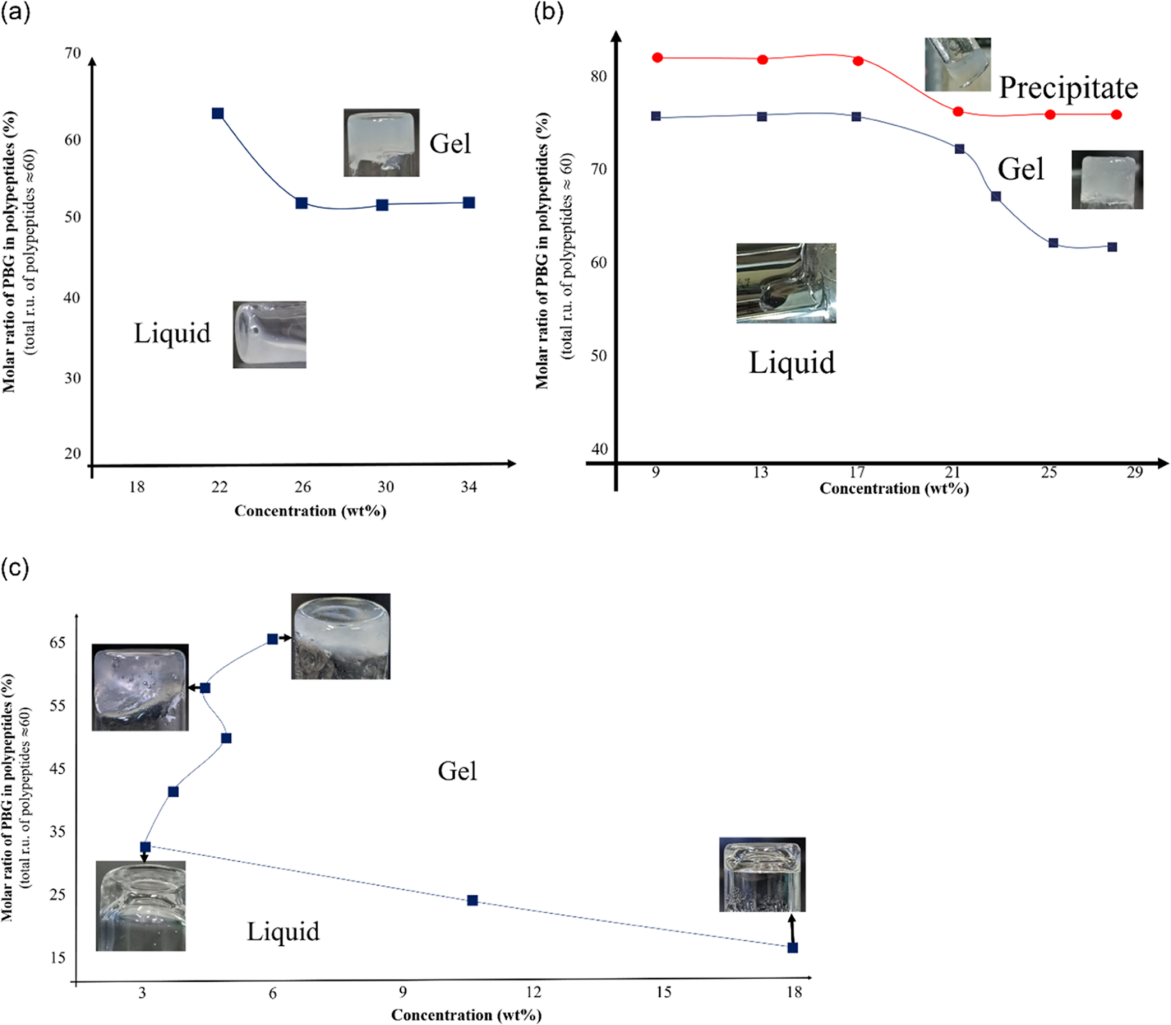
Phase diagrams of (a) K_*y*_-B_*x*_-EG_34_-B_*x*_-K_*y*_, (b) (B_*x*_-*r*-K_*y*_)-EG_34_-(B_*x*_-*r*-K_*y*_), and (c) B_*x*_-K_*y*_-EG_34_-K_*y*_-B_*x*_ copolymers. The molar ratio of PBG in polypeptides is defined as the number of ru of PBG over that of PLL.

The block arrangement of copolymers has profound effects on their gelation behaviors. The tube-inverting method was used to distinguish between the gel state and the sol state. Phase diagrams were constructed for the sol–gel transition for different types and compositions of copolymers and their critical gelation concentrations (CGC) are shown in Figure 5. While reaching the CGC, hydrogels of both types, (B_*x*_-*r*-K_*y*_)-EG_34_-(B_*x*_-*r*-K_*y*_) and K_*y*_-B_*x*_-EG_34_-B_*x*_-K_*y*_, are opaque in appearance, referring to the formation of “agglomerates”. As shown in their phase diagrams (Figure 5a,b), either a higher polymer concentration or a higher molar ratio of PBG leads to a lower CGC of two copolymers. Precipitation occurs when the molar ratio of the hydrophobic segment increases. When the molar ratio of PBG in polypeptides reaches 80% for (B_*x*_-*r*-K_*y*_)-EG_34_-(B_*x*_-*r*-K_*y*_) copolymers, agglomerations are large enough to precipitate out. Hence, the interconnecting gel structure collapses. The morphology that changes with the increase in the molar ratio of PBG can be summarized as clear liquid → opaque gel → blurry liquid with precipitates. Higher gelation concentrations lead to a smaller gelation window (Figure 5b). Precise control of concentration and composition is therefore required for the formation of hydrogels. In other words, the narrow gelation window would limit the ability of such a material to meet multiple requirements (e.g., mechanical properties, porosity, water content, etc.) for biomaterials.

We have identified different appearances and phase diagrams of the penta-block copolymers in aqueous solutions with the same block segments as K_*y*_-B_*x*_-EG_34_-B_*x*_-K_*y*_ but reversing the sequence of B_*x*_ and K_*y*_ to form B_*x*_-K_*y*_-EG_34_-K_*y*_-B_*x*_. With the same polymer concentration and segment length, the K_*y*_-B_*x*_-EG_34_-B_*x*_-K_*y*_ solution is opaque as previously mentioned (Figure 5a), while B_*x*_-K_*y*_-EG_34_-K_*y*_-B_*x*_ can form transparent gels in water (Figure 5c).

**Figure 6:**
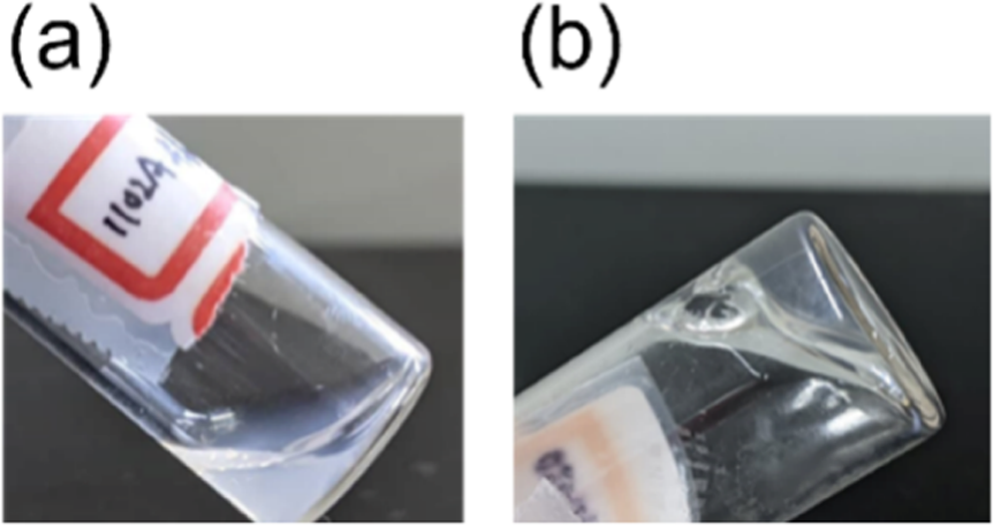
Photos of the morphology of two copolymers (a) K_15_–B_15_-EG_34_-B_15_–K_15_ and (b) B_15_–K_15_-EG_34_-K_15_–B_15_. The former contains micelles in a size large enough to induce Mie scattering, and therefore the solution appears opaque. The latter has a high gelation tendency, appearing as a transparent gel.

Figure 6 shows representative examples of K_15_–B_15_-EG_34_-B_15_–K_15_ opaque solutions and with B_15_–K_15_-EG_34_-K_15_–B_15_ transparent gel. As the polymer concentration reaches 3.7 wt %, the solution becomes a solid-like gel (Figure 5c), which has a porous structure of a microscopic scale network morphology as observed by the scanning electron microscopy (SEM) study (Figure S6). The porous structure is a desired property for tissue engineering to facilitate the access to nutrient flow for cell growth and for drug delivery systems with high efficiency of drug storage. The resultant large gelation window, as shown in Figure 5c, indicates that these hydrogels are potentially applicable for tissue engineering since a wide variety of compositions and polymer concentrations can be tuned to meet the requirements of cell growth. The transparency of the B_15_–K_15_-EG_34_-K_15_–B_15_ hydrogel represents that gelation does not require the connection between micron-scale agglomerations as shown in K_*y*_-B_*x*_-EG_34_-B_*x*_-K_*y*_ copolymers but suggests the nanoscale interplay of hydrophobic–hydrophilic interactions. Small-angle X-ray scattering (SAXS) spectroscopy was hence used to investigate the morphology of these block copolymers, and the outcomes will be discussed in the next section.

**Figure 7:**
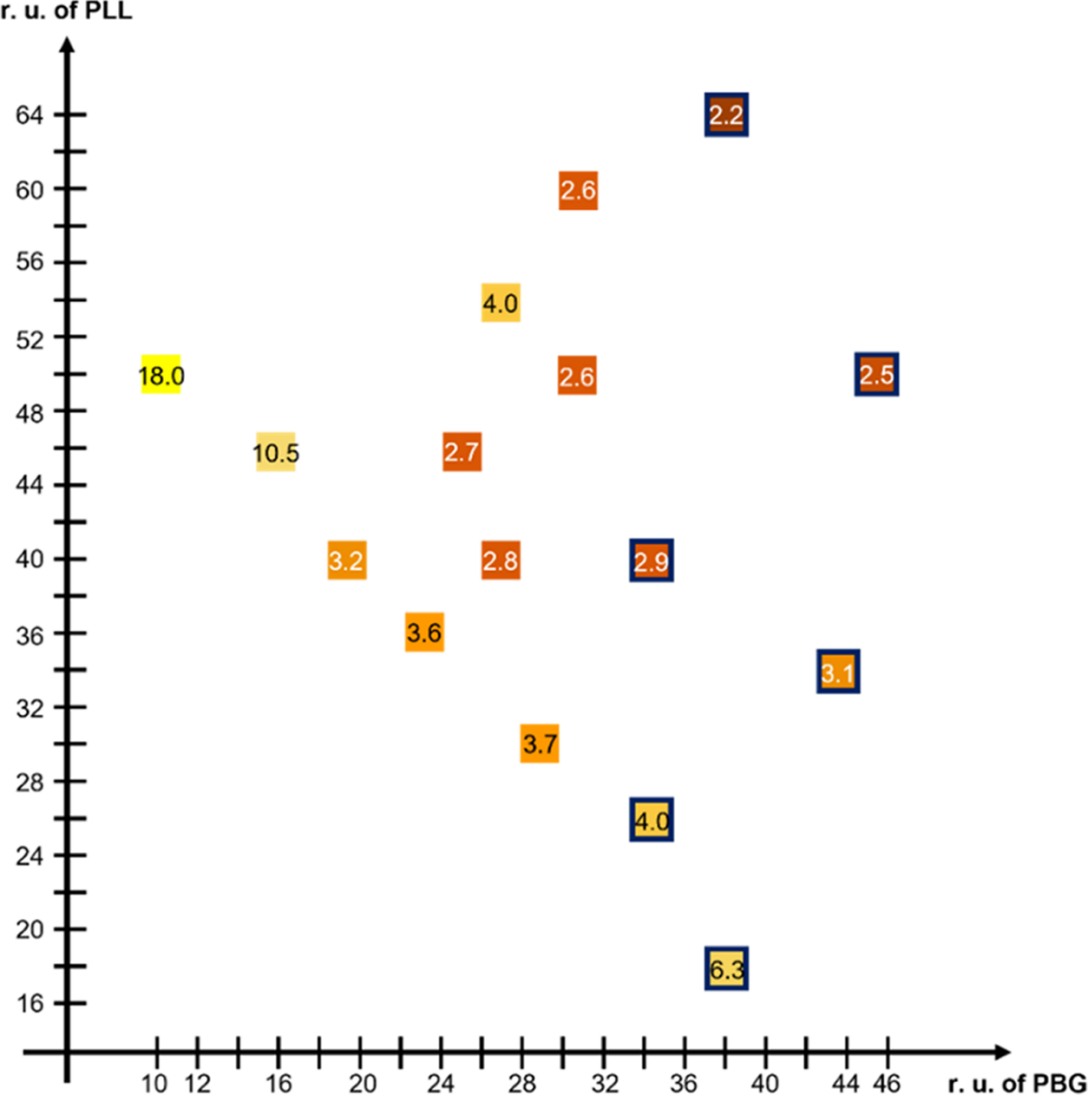
Critical gelation concentration (%) (CGC) of the B_*x*_-K_*y*_-EG_34_-K_*y*_-B_*x*_ copolymer solution of different amounts of repeat units (r.u) in PBG and PLL. The CGC is presented by color from yellow (high CGC) to red (low CGC) for better clarification. Samples with opaque appearance are framed with dark blue.

The effect of the chain lengths of PBG and PLL blocks that constitute the B_*x*_-K_*y*_-EG_34_-K_*y*_-B_*x*_ copolymer on CGC is also investigated as shown in Figure 7. The CGC corresponds to the repeat unit of each block with the x and y axes representing the total repeat units of PBG (2*x*) and PLL (2*y*), respectively. The label color varies from “yellow” to “red”, representing high to low CGC, respectively. This plot provides the lowest solution concentration to fabricate a hydrogel for researchers. The content of this plot is consistent with a previous report by Nowak et al., who showed that the lack and the excess of PLL in poly(lysine-*b*-leucine) copolypeptides cause precipitation and low viscosity solution, respectively, instead of hydrogel formation.^50^

### Nanostructure of Aggregates Resolved by SAXS

To understand the significant extension of the gelation window due to block sequence arrangements, SAXS measurements were performed to examine the nanostructures that lead to gel formation. The ability to measure the morphology of the hydrogel plays a crucial role in optimizing the hydrogel composition and functionality. However, all of the existing characterization methods have inherent limitations in sample preparation, measurement resolution, local variation, and statistical data treatment.^51^ Electron microscopy (called the direct method) provides the real space image with resolution up to the submicrometer scale to the nanoscale, but this technique requires a dehydrated gel (dry sample) with a distorted structure and misleading information. The rapid cooling of cryo-SEM and cryo-TEM causes geometry expansion or gel structural change. On the other hand, the indirect method of X-ray scattering techniques provides real space information by fitting the scattering data with established models. The sample of the scattering method can be used as is, without special preparation. It is a powerful tool to quantitatively measure the nanoscale structure of the hydrogel.

**Figure 8:**
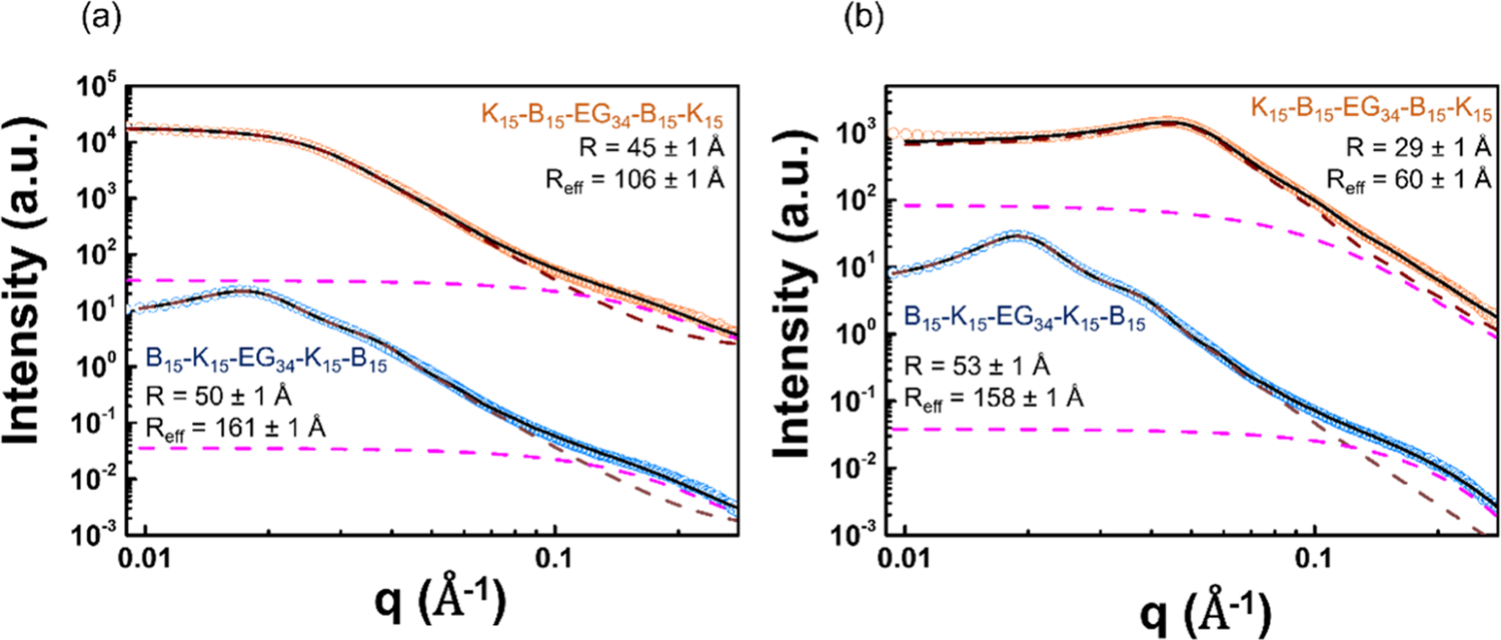
SAXS patterns of (a) 4.0 and (b) 7.0 wt % penta-block copolymers. *R* and *R*_eff_ are the radius and effective radius, respectively. The best-fitted results (black solid curve) are obtained by two spherical models with larger (brown dotted line) and smaller sizes (pink dotted line).

The SAXS patterns of B_15_–K_15_-EG_34_-K_15_–B_15_ and K_15_–B_15_-EG_34_-B_15_–K_15_ (Figure 8) are analyzed using a two-sphere model [eq 1, Experimental Methods], which requires the least number of fitting parameters: the scattering contribution of the larger and smaller sized spheres are presented by the brown and pink dotted lines, respectively. The noticeable broad peak in the low *q* region (<0.03 Å^–1^ for B_15_–K_15_-EG_34_-K_15_–B_15_ and <0.06 Å^–1^ for K_15_–B_15_-EG_34_-B_15_–K_15_) originates from the interparticle interactions between the larger spheres, where a structure factor *S*(*q*), hard-sphere (HS) model [eq 2, Experimental Methods] is incorporated into the larger spherical model.

The smaller spheres (pink dotted line) appear in all samples of both B_15_–K_15_-EG_34_-K_15_–B_15_ and K_15_–B_15_-EG_34_-B_15_–K_15_ series and their radius varies in the range of 10–15.9 Å, indicative of a size similar to that between a unimer and tetramer. This suggests a possible scenario that some molecules could form stable self-assemblies with one or a few (<5) collapsing with each other (or by itself) in both series. It should be noted that the observed initial decay can be assigned to either *q*^–4^ (spheres) or *q*^–2^ (Gaussian chains) due to the absence of SAXS data in a higher *q* range. Therefore, the so-called “smaller spheres” are also possibly contributed by scattering from Gaussian chains, which presumably correspond to the hydrophilic blocks of polymer chains.

For the larger spheres, we found that both B_15_–K_15_-EG_34_-K_15_–B_15_ and K_15_–B_15_-EG_34_-B_15_–K_15_ form aggregates with a similar size (*R* = 50 ± 1 and 45 ± 1 Å, respectively) at 4.0 wt % (Figure 8a). As the polymer concentration increases to 7.0 wt %, the best-fitting *R* of K_15_–B_15_-EG_34_-B_15_–K_15_ decreases by 55% (29 ± 1 Å). On the contrary, the concentration of B_15_–K_15_-EG_34_-K_15_–B_15_ only increases by 6% to the same extent of concentration variation (Figure 8b). The invariant *R* of B_15_–K_15_-EG_34_-K_15_–B_15_ aggregates with concentration indicates the high stability of the larger spherical aggregates. On the other hand, the reduced *R* of K_15_–B_15_-EG_34_-B_15_–K_15_ at 7.0 wt % demonstrates structural variation of the aggregates with concentration. However, the opaque appearance (Figure 6a) suggests the formation of submicron or micron agglomerates in the solution, causing multiple scattering of light, with their size exceeding the SAXS probing range of the minimal *q* value (, where λ and θ are the wavelengths of the incident synchrotron beam and the scattering angle, respectively). We hypothesize that the smaller aggregates are the intermediate structure (or constituents) of the agglomerates. This hypothesis is reflected by the overestimated volume fraction (21%) used in *S*(*q*) (much higher than the real polymer concentration, 4%), implying that the aggregates are subjected to spatial confinement rather than randomly distributed in the whole solution.^52^

**Figure 9:**
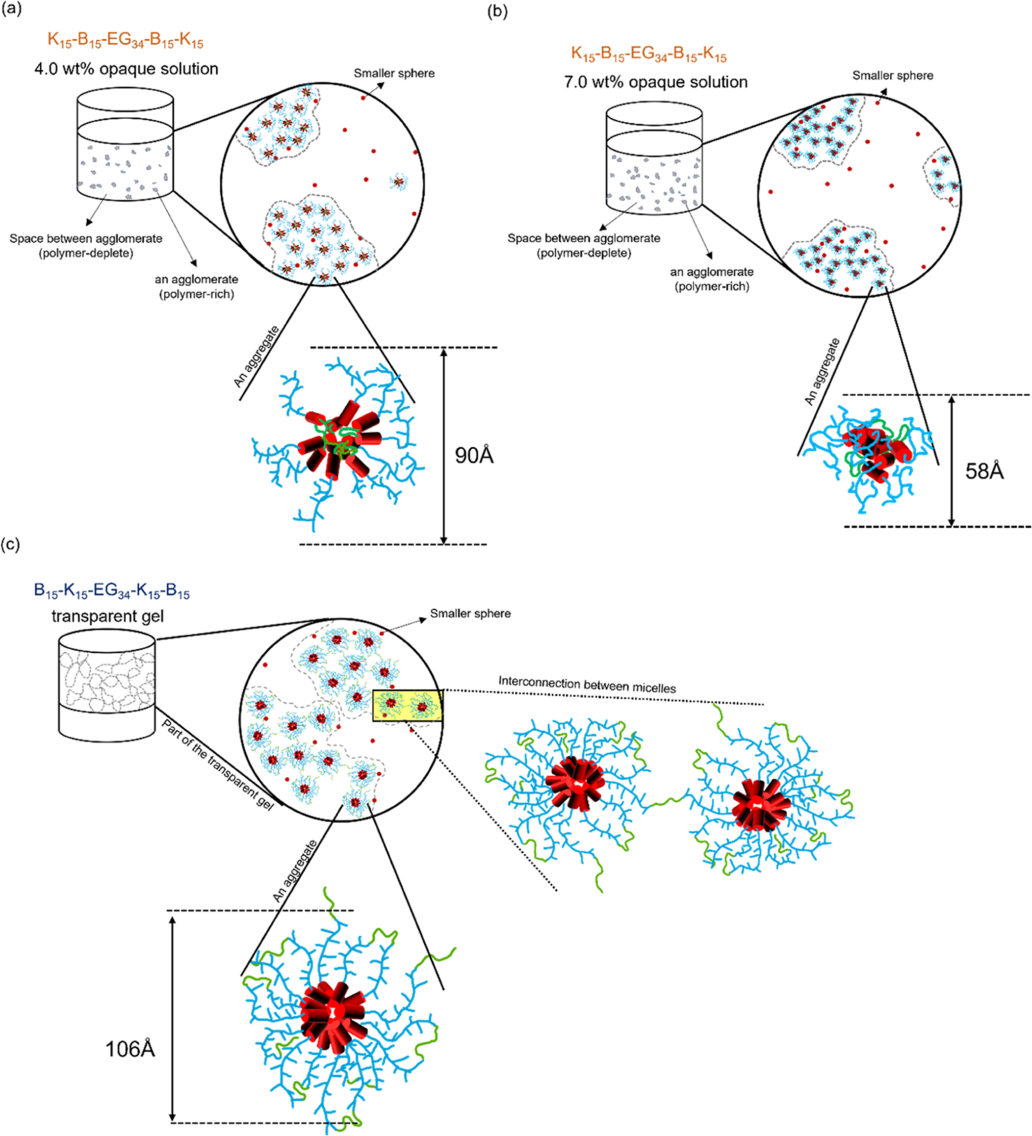
Schematic illustrations of K_15_–B_15_-EG_34_-B_15_–K_15_ opaque solution at concentrations of (a) 4.0 wt % and (b) 7.0 wt %; (c) Clear gel of B_15_–K_15_-EG_34_-K_15_–B_15_. Red rod: PBG segments. Blue branched chain: PLL segments. Green chain: PEG segments. The gray boundary marks the space occupied by each agglomerate.

The agglomerates are polymer-rich regions, rendering large polymer-depleted regions in between (Figure 9a,b), while smaller aggregates are confined in their relative positions within an agglomerate. Judging from the high fluidity of the solution macroscopically, we further hypothesize that the agglomerates possess high mobility in the polymer-depleted region consistent with the fact that the CGC of K_15_–B_15_-EG_34_-B_15_–K_15_ occurs at a polymer concentration (26.0 wt %) much higher than its counterpart, B_15_–K_15_-EG_34_-K_15_–B_15_ (3.7 wt %). Moreover, compared with the opaque appearance of K_15_–B_15_-EG_34_-B_15_–K_15_ resulting from the polymer-rich regime, agglomerations of B_15_–K_15_-EG_34_-K_15_–B_15_ are swelled more by the solvent, leading to a smaller difference in refractive index between the polymer-rich and polymer-deplete regions, resulting in transparent appearance. Further evaluation of the concentration-dependent size of the aggregates obtained from the SAXS measurements on K_15_–B_15_-EG_34_-B_15_–K_15_ solutions provides more insight into the possible morphological variation. Note that the penta-block polymer has the block sequence of hydrophilic (K_15_) – hydrophobic (B_15_) – hydrophilic (EG_34_) – hydrophobic (B_15_) – hydrophilic (K_15_) and the center hydrophilic PEG segment has an estimated radius of gyration of 14 Å, based on free PEG of the same molecular weight.^53^ Bending the relatively short center PEG segment (as the hairpin configuration) to allow the two hydrophobic B_15_ segments of the same chain to self-aggregate is energetically unfavorable. We therefore hypothesize two possible types of aggregations to rationalize the large aggregates observed at 4.0 wt % (*R* = 45 ± 1 Å) and 7.0 wt % (*R* = 29 ± 1 Å). First, the short center PEG segments of several molecules are likely constituting the innermost hydrated “core” of the aggregates presumably with the hydrophobic B_15_ forming an intermediate layer next to the core and the outermost layer of the aggregates is composed of hydrated hydrophilic K_15_ as the zoomed-in aggregate illustrated in Figure 9a. The other possible configuration of the aggregates is having the hydrophobic B_15_ segments from different molecules randomly shielded by hydrophilic K_15_ and PEG with a less defined internal structure of the aggregates as shown in the zoomed-in aggregate illustrated in Figure 9b. The former scenario (Figure 9a) contains well-defined hydrophobic and hydrophilic domains, which are expected to have a similar density to that of the expected hydrophilic/hydrophobic segregation in B_15_–K_15_-EG_34_-K_15_–B_15_ aggregates (explained in the next paragraph). Based on this assumption, we would expect the B_15_–K_15_-EG_34_-K_15_–B_15_ and K_15_–B_15_-EG_34_-B_15_–K_15_ aggregates to have similar sizes in such configurations. Therefore, we presume the 4.0 wt % K_15_–B_15_-EG_34_-B_15_–K_15_ aggregates (*R* = 45 ± 1 Å) should take the well-defined core-to-shell configuration, while the 7.0 wt % K_15_–B_15_-EG_34_ -B_15_–K_15_ aggregates (*R* = 29 ± 1 Å) likely form aggregates with a less organized internal structure. A reasonable explanation is that the higher entropy of smaller aggregates (leading to higher number density) can be promoted by a higher volume fraction of polymers, overcoming the lower energy aggregates with a well-defined internal structure.

We propose a hypothetically different formation mechanism for the stable, well-defined B_15_–K_15_-EG_34_-K_15_–B_15_ aggregates from K_15_–B_15_-EG_34_-B_15_–K_15_ aggregates, though their sizes appear similar to each other. The polymer chain has three consecutive hydrophilic blocks (K_15_ and EG_34_) located at the middle section capped by two hydrophobic blocks. Unlike K_15_–B_15_-EG_34_-B_15_–K_15_, the total length of the middle hydrophilic section of B_15_–K_15_-EG_34_-K_15_–B_15_ is sufficiently long to form flower-like micelles with a hydrophobic B_15_ core and a hydrophilic K_15_-EG_34_-K_15_ shell (Figure 9c). This configuration has been reported in a cholesterol-capped PEG series with a similar block arrangement.^54^ However, the formation of flower-like micelles does not exclude the possible interconnection between micelles with the two hydrophobic B_15_ blocks located at different cores of the adjacent micelles at high concentrations, forming percolation (or network) between micelles. Such a network leads to the gelation of the material. The fact that a nearly constant *R*_eff_ from the scattering structure factor at different polymer concentrations (4.0 and 7.0 wt %) further confirms this hypothesis. The effective volume fractions of 4.0 and 7.0 wt % samples, based on the best-fit hard-sphere structure factor, *S*(*q*), are 22 and 28%, respectively, much higher than the prepared polymer concentrations, further suggesting the formation of agglomerates as observed in the K_15_–B_15_- EG_34_-B_15_–K_15_ series. However, both *R*_eff_ and effective volume fractions are larger than those of the K_15_–B_15_-EG_34_-B_15_–K_15_ series, implying that the agglomerates are likely larger (or more connected to each other) and the individual aggregates within the agglomerates are less heterogeneous to the solvent background than those in the K_15_–B_15_-EG_34_-B_15_–K_15_ series. The former causes fast intensity decay, while the latter yields less refractive index difference between agglomerates and solvent. Both result in a reduction of opaqueness. The gelation of K_15_–B_15_-EG_34_-B_15_–K_15_ presumably follows the same interconnecting mechanism with the B_15_ blocks of the same chain anchoring at different agglomerates.

Tuning segment length of B_*x*_-K_*y*_-EG_34_-K_*y*_-B_*x*_ provides more insight into how the size of the flower-like micelles can be. We design the experiment, where the total number of repeat units of the peptides is fixed to be 60 (i.e., 2*x* + 2*y* = 60) with various ratios of hydrophobic to hydrophilic chain length. At 7.0 wt % concentration, only B_8_–K_23_-EG_34_-K_23_–B_8_ is in solution states, while the others are defined as angle by the tube-inverting method. The SAXS data of four polymers, B_8_–K_23_-EG_34_-K_23_–B_8_, B_10_-K_20_-EG_34_-K_20_-B_10_, B_15_–K_15_-EG_34_-K_15_–B_15_, and B_18_–K_13_-EG_34_-K_13_–B_18_ are analyzed and compared with each other (Figure S7). All of them can be best fitted by a large spherical model in combination with either a small spherical model (as the previous best fitting procedure) or a polymer chain (for smaller units of B blocks) model. Decreasing length of the B blocks expectedly affects the flower-like micelles (the larger aggregates) in two aspects: (1) a smaller size of the hydrophobic core and (2) a lower probability for the B blocks to perform the interconnection of the micelles. The former is consistent with the trend of decreasing radii of flower-like micelles, while the latter is reflected in the solution behavior of the B_8_–K_23_-EG_34_-K_23_–B_8_ sample. Since the reduced r.u. in the B blocks renders higher solubility of the polymer, we have found the polymer scattering model to account for better fits of smaller objects for the B_8_–K_23_-EG_34_-K_23_–B_8_ and B_10_-K_20_-EG_34_-K_20_-B_10_ solutions (the pink dotted line in Figures 8 and S7). For B_*x*_-K_*y*_-EG_34_-K_*y*_-B_*x*_ samples with longer B blocks, the polymer model has to be replaced by a small spherical model to yield better fits because the high *q* intensity scaling varies from *I ∼ q*^–2^ to *q*^–4^. The formation of smaller aggregates with a radius ranging from 7 to 12 Å can be rationalized by the collapse of a few polymer chains. To evaluate how the r.u. of the hydrophilic end groups (K blocks) of K_*y*_-B_*x*_-EG_34_-B_*x*_-K_*y*_ affects the system, we synthesized three K_*y*_-B_*x*_-EG_34_-B_*x*_-K_*y*_ copolymers with different PLL lengths: K_15_–B_15_-EG_34_-B_15_–K_15_, K_27_–B_15_-EG_34_-B_15_–K_27_, and K_44_–B_16_-EG_34_-B_16_–K_44_. Although the appearance of the samples becomes more transparent with increasing length of the K block, there is no gelation in the samples (Figure S8). The higher transparency for samples with longer K blocks suggests the reduced quantity of large agglomerates. It is expected that the overextended end K blocks lead to two major effects on aggregation: higher solubility and more significant steric hindrance between large aggregates. The former would change the small aggregates into an extended coil, while the latter effectively inhibits the interconnection of large aggregates into agglomerates causing no gelation at low polymer concentrations (at 7.0 wt %).

The experimental outcomes suggest a few important strategies for forming a stable low-concentration hydrogel with multiblock (at least triblock) polypeptides. First, stable hydrophobic cores are required. This can be easily achieved by having hydrophobic end groups to form flower-like micelles with hydrophobic cores (as “connection” points for the network). The middle hydrophilic block(s) requires sufficient length for the hydrophobic ends to adopt a U-shape forming the core. Some of the chains with only one end anchoring at a core can extend to another micellar core, resulting in an interconnection between micelles. We expect that similar copolymers with such a block arrangement and similar chain length would lead to a gel structure as well. We affirm this claim by showing a new block copolymer with a reduced chain length of PEG and an increased length of PLL forming a gel structure at a low polymer concentration. We took the diamine with two repeat units of ethylene glycol, 2,2′-(ethylene dioxy) bis(ethylamine) (EDEA), as the initiator instead of PEG_34_-(NH_2_)_2_. The copolymer possesses approximately the same PBG wt % in the polymer with the B_15_–K_15_-EG_34_-K_15_–B_15_ copolymer to retain the hydrophobic to hydrophilic ratio within the copolymer. The resultant product, B_14_–K_19_-EG_2_-K_19_–B_14_, indeed forms a gel easily, with CGC as low as 2.3 wt %. The sample appears gel-like and transparent from 2.3 to 10.0 wt % (Figure S9). Comparing such a sample with B_15_–K_15_-EG_34_-K_15_–B_15_, which has the same PBG wt % and the same molecular weight, we observed that both exhibit clear appearance and low CGC, concluding that the replacement of ethylene glycol with lysine does not disrupt the self-assembly structure and gel formation. We speculate that the repulsion force arising from the positive charge of PLL contributes significantly to the stability of the flower-like micelle structures. This hypothesis is substantiated by experiments involving the addition of salt to the solution. When deionized water is replaced by 1 wt % NaCl aqueous solution (left vial), the gel structure experiences a collapse, providing tangible evidence of the influence of the PLL charge on the self-assembly dynamics and stability of the micelle structures as shown in Figure S10. The B_*x*_-K_*y*_-EG_2_-K_*y*_-B_*x*_ gel can be a promising material for supporting nerve regeneration. The merit of B_*x*_-K_*y*_-EG_2_-K_*y*_-B_*x*_ lies in the fact that the number of ethylene glycol units can also be tuned in a wide range. A wide gelation window is expected, consequently, and there is high flexibility in tuning the composition for optimizing gel mechanical properties and cell viability.

## Conclusions

We have reported micellar formation and gelation of a novel penta-block copolymer. As expected, the block sequence and hydrophilic/hydrophobic length ratio of copolymers significantly affect their self-assembly architecture and gelation window. Random copolymer (B_*x*_-*r*-K_*y*_)-EG_34_-(B_*x*_-*r*-K_*y*_) and hydrophilic block ending copolymer K_*y*_-B_*x*_-EG_34_-B_*x*_-K_*y*_ do not form transparent hydrogels at a low concentration (≤22 wt %), while B_*x*_-K_(30-x)_-EG_34_-K_(30-x)_-B_*x*_ forms hydrogel when 10 ≤ *x* ≤ 18 at a concentration of 7.0 wt %. The formation of the hydrogel presumably requires hydrophobic “clusters” as gelation points connected by a water-rich hydrophilic network throughout the sample. For K_*y*_-B_*x*_-EG_34_-B_*x*_-K_*y*_, the short central hydrophilic PEG segment leads to less defined micelles, which further aggregate into large agglomerates with a size in the range of wavelengths of visible light. On the contrary, B_*x*_-K_*y*_-EG_34_-K_*y*_-B_*x*_ forms well-defined flower-like micelles, and these micelles further interconnected with each other to form agglomerates until gelation. Transparency of the gel indicates that the polymer chains are well-swollen by water with a less refractive index difference between agglomerates and water and a larger agglomerate size exceeding the interfering length scale of light. We conclude that a long enough hydrophilic central segment is critical for amphiphilic multiblock copolymers to form flower-like micelles and low-concentration gel, thereby opening a wider gelation window. With the outstanding effect on stimulating neuron growth of PBG, the materials have great potential for applications in neural tissue engineering. The micellar shape with a hydrophobic center may also promote sustained drug release to stimulate neuron growth on the scaffold, which is left for future investigation. The nanoscale structural investigation provides insight into the rational design for block sequence and block length of multiblock copolymers to form the hydrogel.

## Experimental Methods

### Chemicals

The following chemicals were used as received: triphosgene (Sigma Aldrich), N^ε^-*tert*-butyloxylcarbonyl-l-lysine (Fluorochem), l-glutamic acid γ-benzyl ester (Fluorochem), PEG (Acros), chlorotrimethylsilane (TMSCl, Sigma Aldrich), 4-toluenesulfonyl chloride (p-TsCl, Sigma Aldrich), sodium azide (NaN_3_, Sigma Aldrich), ethyl acetate (EA, Macron Fine Chemicals), methanol (MeOH, Macron Fine Chemicals), chloroform (Duksan), trifluoroacetic acid (TFA, Fluorochem), hexane (Macron Fine Chemicals), dichloromethane (DCM, Macron Fine Chemicals), and diethyl ether (Macron Fine Chemicals). Triethylamine (TEA, Sigma), 2, 2′-(ethylene dioxy) bis(ethylamine) (EDEA, Sigma Aldrich), *N*,*N*-dimethylformamide (DMF, Sigma), and tetrahydrofuran (THF, Macron Fine Chemicals) were distilled before used.

### Instruments for Characterization

Chemical structures of copolymers were characterized by NMR spectroscopy (Bruker 400 MHz), FT-IR spectroscopy (Spectrum 100 and FT/IR 6700), GPC (Jasco PU-2080 Plus HPLC pump/RI-2031 Plus detector/Shodex GPC KD 803 column (8.0 mm × 300 mm)), and MALDI-ToF-MS (Bruker Daltonics Autoflex Speed MALDI-ToF). NMR spectroscopy was utilized for the characterization of all chemicals mentioned. The scanning range of FT-IR expanded from 4000 to 650 cm^–1^. In the GPC experiment, 3 mg of dry polymer was dissolved in 500 μL of HPLC-grade DMF. Only the samples before deprotection were used for measurement to avoid sample clogging in the column. The RI detector, which showed better resolution for our polymers, was used. In the experiment of MALDI-ToF-MS, the sample solution was made from 10 mg of sample in 1 mL of mixed solvent of THF and MeOH. Ten mg α-cyano-4-hydroxycinnamic acid (CHCA) or sinapic acid (SA) was dissolved in the mixed solvent of 0.1% TFA, 50% H_2_O, and 50% acetonitrile as the matrix solution. All solvents used in MALDI-ToF-MS were spectrophotometry grade.

Polymer hydrogels were lyophilized with a freeze-dryer (Kingmech Scientific) to maintain their structure before microscopic observation with SEM (JEOL JSM6510). Samples were coated with platinum for the observation. A 0.01 wt % sample in deionized water was used for most of the Circular Dichroism (CD) measurements to prevent signal saturation. The range of the spectrum spanned from 300 to 170 nm with the step of 0.1 nm. The temperature is kept constant at 25 °C. J-810 (Jasco) kindly provided by Prof. Chao-Tsen Chen’s lab, Department of Chemistry, National Taiwan University was utilized. The transmission small-angle X-ray scattering (SAXS) spectroscopy was carried out at the 23A1 workstation in the National Synchrotron Radiation Research Center (NSRRC, Taiwan). A sample-to-detector distance of 3.875 m, a beam energy of 15 keV, and *q* (defined as , where λ and θ are the wavelength of synchrotron and the scattering angle, respectively) ranging from 0.005 to 0.35 A^–1^ were used. Samples were stacked between two Kapton slides and fixed on the holder.

### Synthesis of Copolymers

The synthesis and characterization of monomers, γ-benzyl glutamate-*N*-carboxyanhydride (BzGlu-NCA) and N^ε^-*tert*-butyloxylcarbonyl-l-lysine-*N*-carboxyanhydride (BocLys-NCA), and the macroinitiators, poly(ethylene glycol) bis(amine) (PEG-(NH_2_)_2_) utilizing reported methods^30,55^ are shown in the Supporting Information. The polymerization of (B_*x*_-*r*-K_*y*_)-EG_34_-(B_*x*_-*r*-K_*y*_) was conducted by simultaneously reacting two types of monomers with PEG-(NH_2_)_2_ as the macroinitiator. The synthesis of B_*x*_-K_*y*_-EG_34_-K_*y*_-B_*x*_, B_*x*_-K_*y*_-EG_2_-K_*y*_-B_*x*_, and K_*y*_-B_*x*_-EG_34_-B_*x*_-K_*y*_ copolymers was carried out by two-step polymerization via sequential monomer addition. Details are also described in the Supporting Information.

### Hydrogel Fabrication

Polymers dissolved in water were sonicated overnight to ensure they were fully mixed, obtaining either fluid sol or a solid gel. The solution was then kept in a 4 °C fridge before the characterization and measurements.

### Phase Diagram

The sol–gel transition is investigated by the tube-inverting method. A vial (diameter = 11 mm) containing well-mixed polymer with water is vertically inverted for 2 min for distinguishing between fluid-state samples and gel-state samples. The solution will be considered in a fluid state if it flows from the top within 2 min of inversion.

### Spherical SAXS model^56^

Our amphiphilic block copolymers can be described by the two spherical models as shown in Figures 8 and S7.
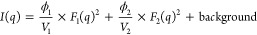
where
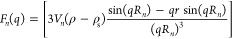
1where *n* is either 1 (larger spheres) or 2 (smaller spheres),  is the volume of the whole particle, *R*_*n*_ is the spherical radius of group *n*, ρ and ρ_s_ are the scattering length density of the particle and solvent, respectively.

### Hard-Sphere SAXS Model^57^

This model is used to describe the interparticle structure factor for spherical particles interacting through hard-sphere interactions. The hard-sphere model uses the Percus–Yevick closure where the interparticle potential is
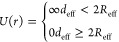
2where *d*_eff_ is the average distance between the centers of the spheres with an effective radius, *R*_eff_ and *d*_eff_ is derived from the effective volume of the spheres, ϕ_eff_, for which we assume the sample to be completely homogeneous (mean field theory).

## Supporting Information Available

Additional synthesis methods, NMR, FT-IR, CD, and SAXS spectra, and SEM image (PDF)
