# Supplementary material for: Block Sequence Effects on the Self-Assembly Behaviors of Polypeptide-Based Penta-Block Copolymer Hydrogels
Source: ACS Appl Mater Interfaces. 2024 Jan 30;16(5):6674–86. doi: 10.1021/acsami.3c18954 (PMC10859891; doi:10.1021/acsami.3c18954)
Supplement: Supplementary file 1 — am3c18954_si_001.pdf [file am3c18954_si_001.pdf]

Supporting Information

Block Sequence Effects on the Self Assembly Behaviors of Polypeptide-Based Penta-Block Copolymer Hydrogels

*Ke-Hsin Wang[1†], Chung-Hao Liu[2], Dun-Heng Tan[1], Mu-Ping Nieh[2,3]*and Wei-Fang Su[1,4]**

[1]Department of Materials Science and Engineering, National Taiwan University, No. 1, Sec. 4, Roosevelt Road, Taipei 10617, Taiwan

[2]Polymer program, Institute of Materials Science, University of Connecticut, 25 King Hill Road, Unit 3136, Storrs, CT-06269-3136, U.S.A.

[3]Department of Chemical and Biomolecular Engineering, University of Connecticut, Storrs, CT 06269, U.S.A.

[4]Department of Materials Engineering, Ming-Chi University of Technology, 84 Gungjuan Rd., Taishan Dist., New Taipei City 243303, Taiwan

† Pritzker School of Molecular Engineering, The University of Chicago, 5640 South Ellis Avenue, Chicago, IL 60637, U.S.A.

* E-mail: suwf@ntu.edu.tw (W.-F. Su). * mu-ping.nieh@uconn.edu (M.-P. Nieh).

Synthesis of macro-initiator

10 g of PEG (Mw: 1500g/mol, 1.0 eq.) was mixed with 4.45 g TEA (6.6 eq.) in 60 mL DCM in an ice bath under N_2. Then 12.71 g p-TsCl (10 eq.) in 70 mL DCM was added to the former solution dropwise through the syringe. After 4 hours of reaction, the yellowish solution was extracted against water twice, followed by removing the majority of organic solvent with the rotary evaporator. Afterward, this concentrated mixture was dropped slowly in cold ether to precipitate out the product, and the excess solvent was removed through decantation. This step was repeated until only white powder was obtained in the flask. At last, excess ether was removed through the rotary evaporator and under high vacuum. The resulting product, PEG-$(OTs)_2$, appeared viscous and white (**Figure S 1a**).

The reaction of PEG-$(OTs)_2$ with NaN_3 to obtain PEG-$(N_3)_2$ was conducted under N_2 at 60°C. 10 g of PEG-$(OTs)_2$ (1.0 eq.) was dissolved in anhydrous DMF (150 mL), followed by the addition of 7.20 g NaN_3 (20 eq.). After 1 hour of reaction, DMF was removed by distillation. The resulted mixture was extracted with brine. The organic layer was collected, concentrated through the rotary evaporator, and the product was dried in *vacuo*. The obtained product was a white solid, PEG-$(N_3)_2$. (yield: 75%, **Figure S 1b**).

In the final step, 300 mg PEG-$(N_3)_2$ (1.0 eq.) was dissolved in THF (3 mL). 262 mg PPh_3 (5.0 eq.) in THF (2 mL) was added slowly through the syringe, followed by few drops of distilled water. The reaction occurred at room temperature for about 8 hours until the azide peak disappeared, as indicated by ^1H NMR. Afterward, the solution was concentrated with the rotary evaporator, and slowly dropped into cold ether for precipitation. Excess solvent was removed by decantation. Precipitation and decantation procedures are repeated until ^1H NMR indicated that PPh_3 was removed. The white powder was obtained at the end and kept in a 4°C fridge before being used. (yield: 80%, **Figure S 1c**)

The corresponding characteristic ^1H NMR chemical shifts (400 MHz, δ) shown in **Figure S 2a** are listed in below. PEG-$(NH_2)_2$ (400MHz, 25˚C, chloroform-*d*): 2.86 (t, 4H), 3.51 (t, 4H), 3.64 (broad, 134H).

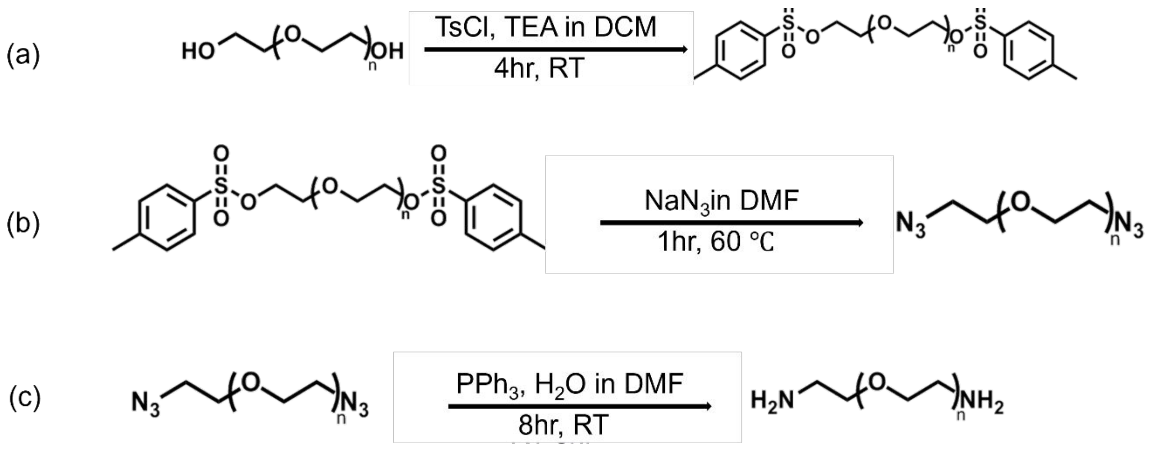

Figure S 1 Synthetic scheme of (a) PEG-$(OTs)_2$, (b) PEG-$(N_3)_2$, and (c) PEG-$(NH_2)_2$.

Synthesis of N-carboxy anhydrides

2.5 g triphosgene was added to the suspension of 4.0 g L-glutamic acid γ-benzyl ester in EA (120 mL) at an elevated temperature of 105℃ for 2 hours. The product was recrystallized in hexane three times for purification. (yield: 70%)

The same method was applied to synthesize BocLys-NCA except for that the anhydrous THF was used as the solvent and the reaction took place at room temperature. Transparent crystals were obtained after the product was dried in a 40℃ oven overnight. (yield: 60%)

The characteristic ^{1}H NMR chemical shifts (400MHz, 25˚C, δ) shown in **Figure S 2b** and **Figure S 2c** are listed in below. BzGlu-NCA (chloroform-*d*): 2.09-2.32 (m, 2H), 2.60 (t, 2H), 4.37 (t, 1H), 5.14 (s, 2H), 6.48 (s, 1H), 7.36 (m, 5H). BocLys-NCA (DMSO-d_6): 1.21-1.73 (m, 15H), 2.90 (q, 2H), 4.42 (t, 1H), 6.67 (broad, 1H), 9.06 (broad, 1H). The characteristic FT-IR (KBr) peaks of BocLys-NCA in **Figure S 4a** and those of BzGlu-NCA in **Figure S 4b** are listed in below. BocLys-NCA: 1821 cm^{-1} (C=O, anhydride), 1762 cm^{-1} (C=O, anhydride), 1691 cm^{-1} (C=O, Boc protecting group). BzGlu-NCA: 1844 cm^{-1} (C=O, anhydride), 1782 cm^{-1} (C=O, anhydride), 1705 cm^{-1} (C=O, ester), 696 cm^{-1}, 506 cm^{-1} (C-H, aromatic).

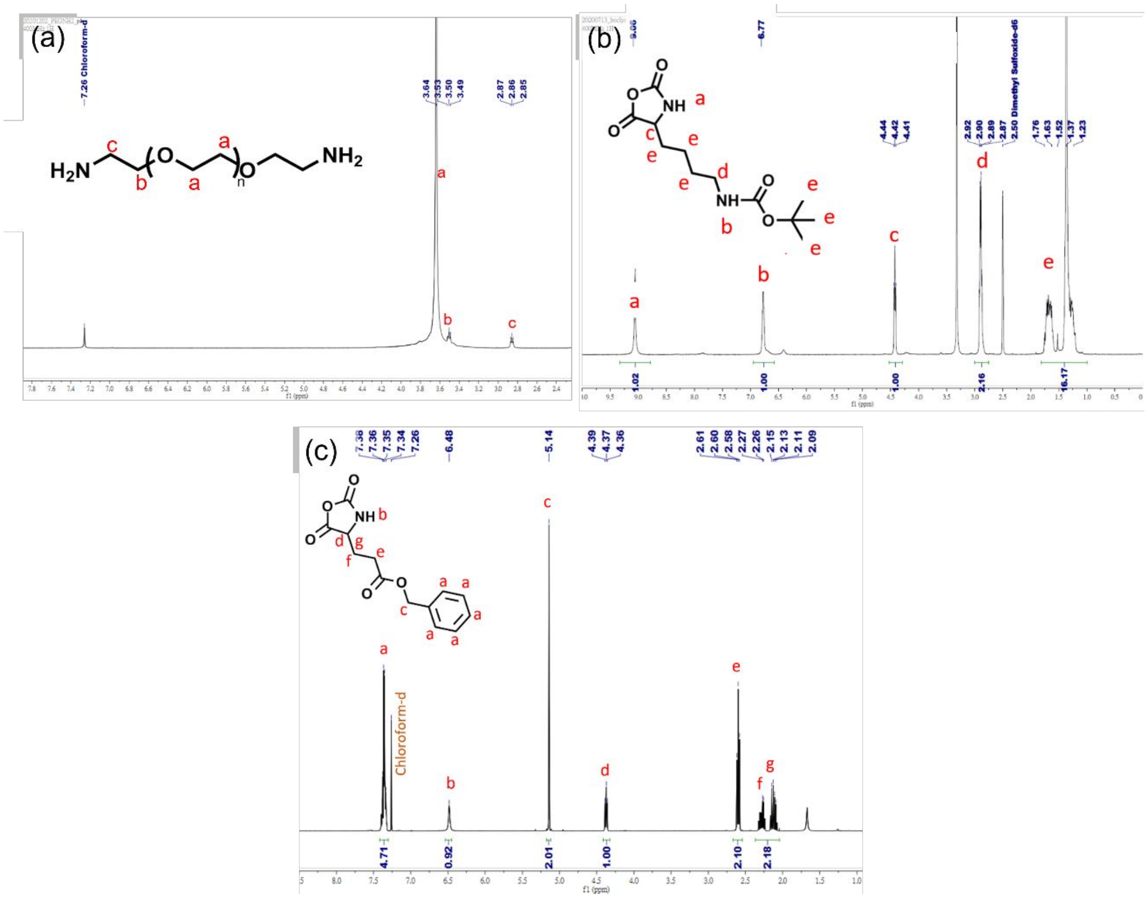

Figure S 2 ^{1}H NMR (400MHz) spectra of (a) macroinitiator PEG-$(NH_2)_2$, (b) BocLys-NCA, and (c) BzGlu-NCA.

Synthesis of (B_x-r-K_y)-EG_{34}-(B_x-r-K_y) copolymer

The polymerization was conducted by simultaneously reacting two types of monomers using PEG-$(NH_2)_2$ macro-initiator. BzGlu-NCA and BocLys-NCA underwent ring-opening polymerization at 0°C for 30 hours in anhydrous DMF. Side reactions were suppressed by the low temperature and the nitrogen flow as strong as 1 mL/s. The solvent was removed by distillation. Re-dissolving the product in little chloroform, precipitating in cold ether, and filtering resulted in the final product as white powder.

Afterward, 1 g of the copolymer was dissolved in 20 mL DCM, followed by the addition of 20 mL TFA for the deprotection reaction to start. 2 hours later, DCM and TFA were mainly removed by the rotary evaporator. The remaining clear sticky solid was then dissolved in MeOH before being poured into the dialysis bag (Mw cut off: 3000). The dialysis buffer was composed of MeOH:H_2O (8:2) at first, while it was replaced by buffer with the ratio of MeOH to H_2O gradually decreasing until pure H_2O was used to replace MeOH as the solvent. Freeze drying of the mixture gave the final product a white solid. ^{1}H NMR chemical shifts are shown in **Figure S 3c**.

Synthesis of B_x-K_y-EG_{34}-K_y-B_x and K_y-B_x-EG_{34}-B_x-K_y copolymer

During the synthesis of B_x-K_y-EG_{34}-K_y-B_x, BzGlu-NCA was added after BocLys-NCA had been fully polymerized by PEG-$(NH_2)_2$, which was determined by FT-IR. On the other hand, BocLys-NCA was added after BzGlu-NCA had been fully polymerized in the case of K_y-B_x-EG_{34}-B_x-K_y copolymer. The polymerization conditions and the deprotection method were identical to that of (B_x-r-K_y)-EG_{34}-(B_x-r-K_y) copolymer. The ^{1}H NMR chemical shifts of copolymers are at the same position as those of (B_x-r-K_y)-EG_{34}-(B_x-r-K_y) copolymer (**Figure S 3**).

Synthesis and ^{1}H NMR of B_{14}-K_{19}-EG_2-K_{19}-B_{14} copolymers

EDEA was used as the initiator to replace PEG as compared with the synthesis of B_x-K_y-EG_{34}-K_y-B_x. ^{1}H NMR (400MHz, 25°C, trifluoroacetic acid-d, δ) chemical shifts are listed: 1.77-2.41 (broad, 284 H), 2.70-2.84 (broad, 56H), 3.46 (broad, 76 H), 3.84 (s, 4H), 4.04-4.10 (broad, 8H), 4.80 (s, 38 H), 4.89 (s, 28 H), 5.30-5.43 (q, 56 H), 7.47 (broad, 140 H).

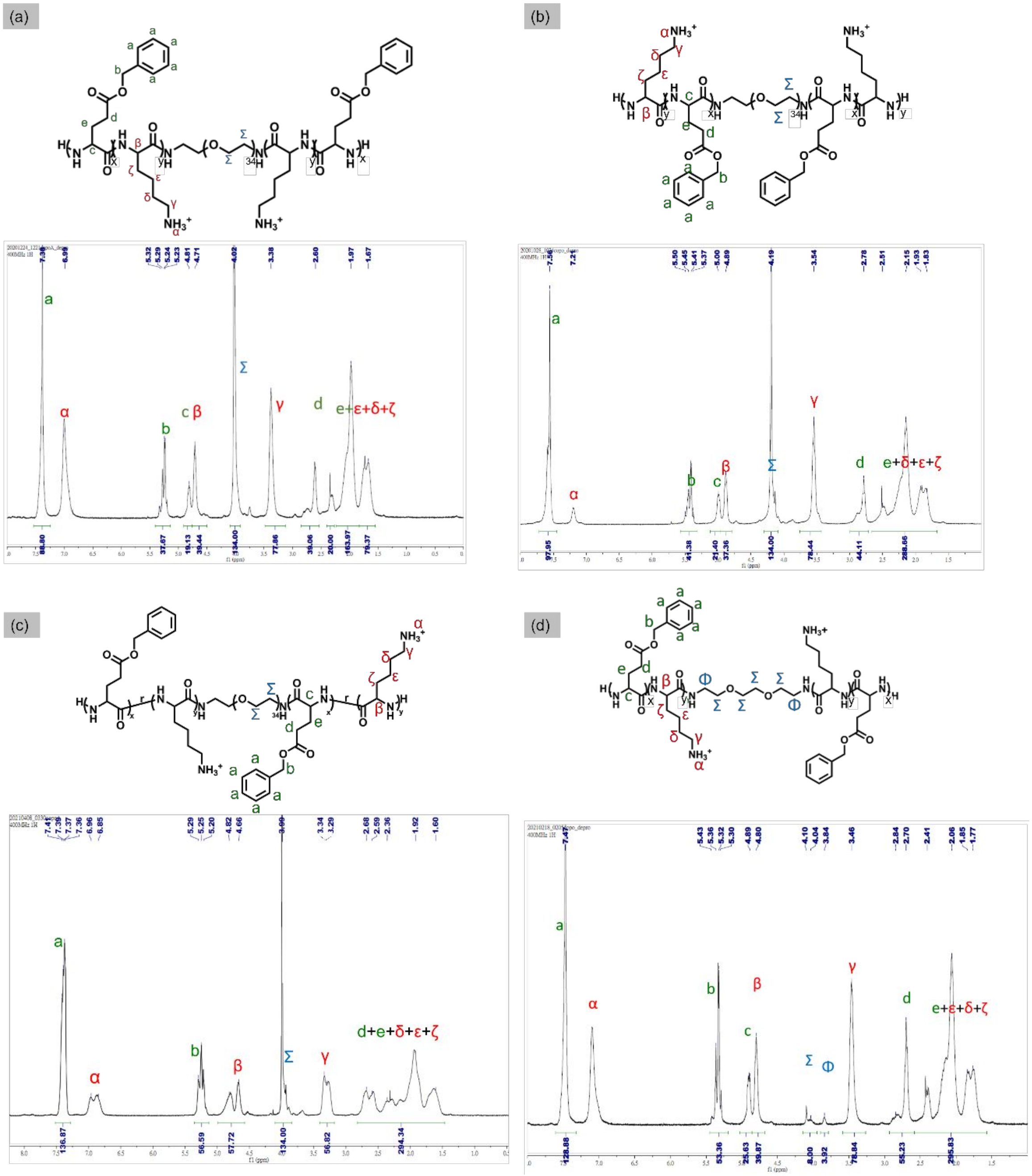

Figure S 3 ^{1}H NMR (400MHz) spectra of (a) B_{10}-K_{20}-EG_{34}-K_{20}-B_{10}, (b) K_{20}-B_{11}-EG_{34}-B_{11}-K_{20}, (c) (B_{14}-r-K_{14})-EG_{34}-(B_{14}-r-K_{14}) (d) B_{14}-K_{19}-EG_2-K_{19}-B_{14} penta-block copolymers. (trifluoroacetic acid-d, 400 MHz)

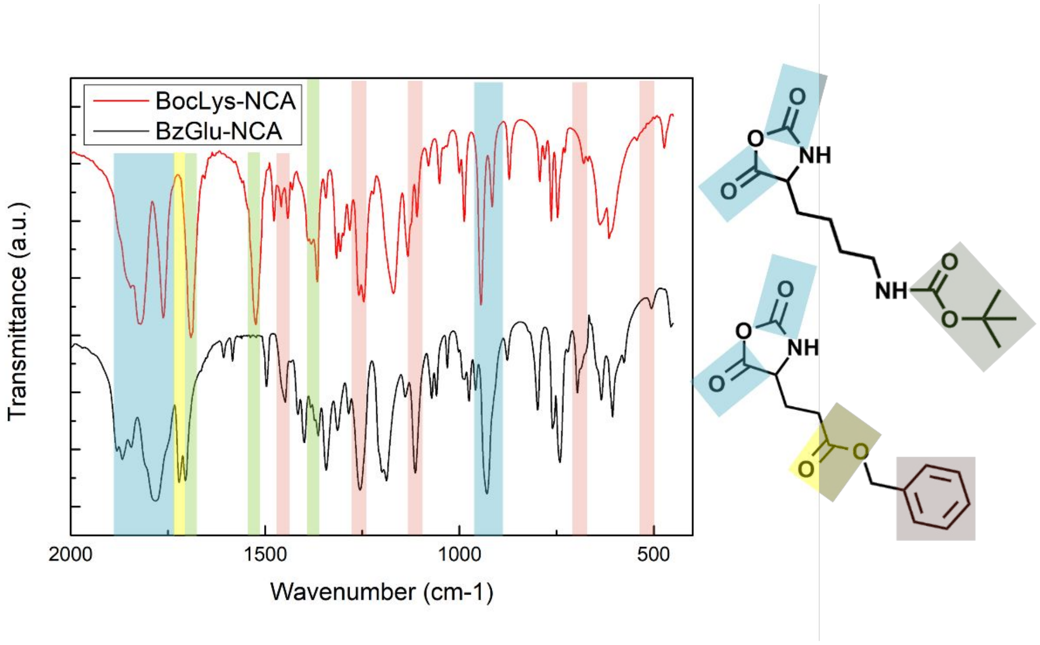

Figure S 4 FT-IR spectra of BocLys-NCA (red line) and BzGlu-NCA (black line). Contribution of different groups are differentiated with labeled colors.

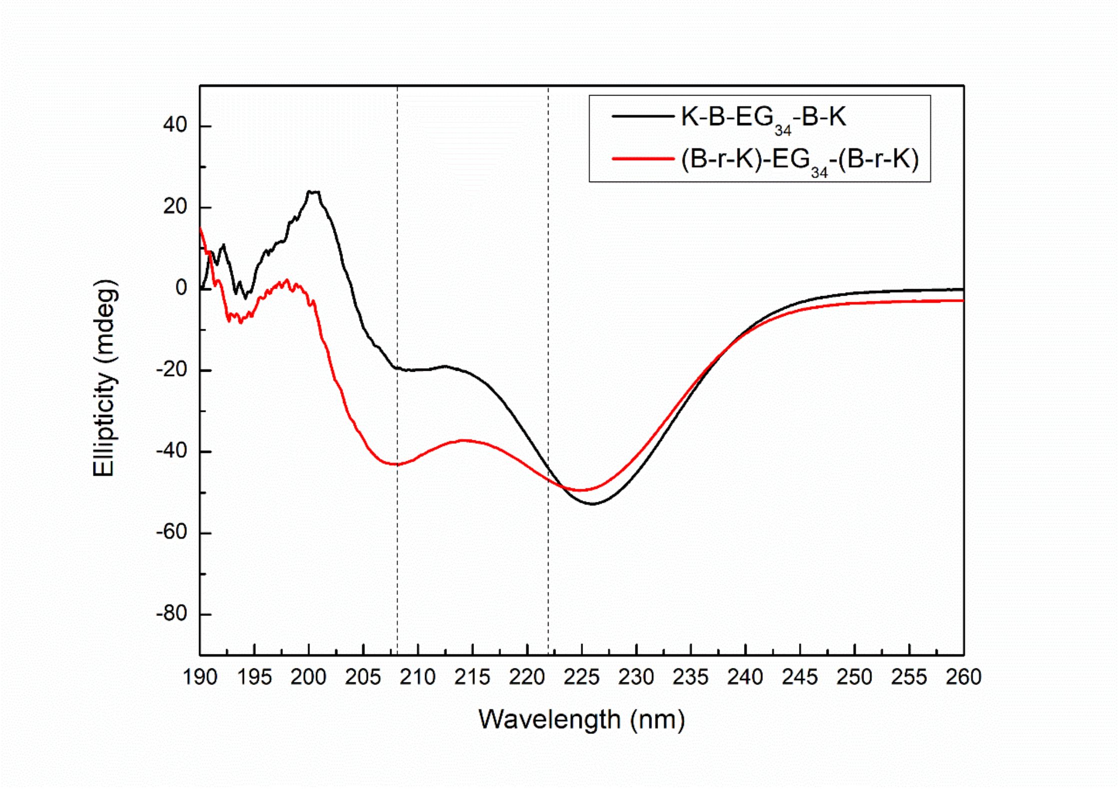

Figure S 5 CD spectra of K_{15}-B_{15}-EG_{34}-B_{15}-K_{15} and (B_{14}-r-K_{14})-EG_{34}-(B_{14}-r-K_{14}). Dash vertical lines indicates the positions of standard α-helix peaks, at 208 nm and 222 nm. Peaks of K_{15}-B_{15}-EG_{34}-B_{15}-K_{15} are more distorted and red shifted, indicating the present of aggregation.

Table S 1 Comparison between feed ratio of copolymers and the experimental numbers of repeat unit after polymerization.

Copolymer	Feed ratio in molar before copolymerization			Chemical composition of copolymer in molar after copolymerization i)			Mn i)
	PEG	BzGlu-NCA	BocLys-NCA	Number of EG r. u.	Number of B r. u.	Number of K r. u.	-
$(B_{13}$-r-$K_{18})$-EG_{34}-$(B_{13}$-r-$K_{18})$	1	25	35	34	25	36	11583
$(B_{14}$-r-$K_{14})$-EG_{34}-$(B_{14}$-r-$K_{14})$	1	30	30	34	28	28	11216
$(B_{17}$-r-$K_{13})$-EG_{34}-$(B_{17}$-r-$K_{13})$	1	35	25	34	33	25	11927
$(B_{19}$-r-$K_{12})$-EG_{34}-$(B_{19}$-r-$K_{12})$	1	38	22	34	37	23	12547
$(B_{20}$-r-$K_{10})$-EG_{34}-$(B_{20}$-r-$K_{10})$	1	40	20	34	39	20	12601
$(B_{22}$-r-$K_{9})$-EG_{34}-$(B_{22}$-r-$K_{9})$	1	43	17	34	44	18	13440
$(B_{22}$-r-$K_{8})$-EG_{34}-$(B_{22}$-r-$K_{8})$	1	45	15	34	43	15	12837
$(B_{25}$-r-$K_{5})$-EG_{34}-$(B_{25}$-r-$K_{5})$	1	50	10	34	49	10	13511
K_{24}-B_{8}-EG_{34}-B_{8}-K_{24}	1	15	45	34	16	47	11020
K_{20}-B_{11}-EG_{34}-B_{11}-K_{20}	1	20	40	34	21	39	11091
K_{16}-B_{13}-EG_{34}-B_{13}-K_{16}	1	27	33	34	26	32	11290
K_{15}-B_{15}-EG_{34}-B_{15}-K_{15}	1	30	30	34	30	30	12129
K_{10}-B_{21}-EG_{34}-B_{21}-K_{10}	1	40	20	34	41	20	13039
K_{27}-B_{15}-EG_{34}-B_{15}-K_{27}	1	30	60	34	30	54	14982
K_{44}-B_{16}-EG_{34}-B_{16}-K_{44}	1	30	90	34	33	88	19991

B_5-K_{25}-EG_{34}-K_{25}-B_5	1	10	50	34	9	50	9871
B_8-K_{23}-EG_{34}-K_{23}-B_8	1	15	45	34	15	46	10673
B_{10}-K_{20}-EG_{34}-K_{20}-B_{10}	1	20	40	34	19	39	10653
B_{12}-K_{18}-EG_{34}-K_{18}-B_{12}	1	25	35	34	24	35	11236
B_{15}-K_{15}-EG_{34}-K_{15}-B_{15}	1	30	30	34	30	30	11910
B_{18}-K_{13}-EG_{34}-K_{13}-B_{18}	1	35	25	34	35	26	12493
B_{20}-K_{10}-EG_{34}-K_{10}-B_{20}	1	40	20	34	40	20	12820
B_{14}-K_{27}-EG_{34}-K_{27}-B_{14}	1	20	50	34	28	54	14544
B_{14}-K_{20}-EG_{34}-K_{20}-B_{14}	1	25	35	34	27	40	12533
B_{20}-K_{20}-EG_{34}-K_{20}-B_{20}	1	40	40	34	39	40	15161
B_{18}-K_{32}-EG_{34}-K_{32}-B_{18}	1	40	60	34	36	63	17448
B_{13}-K_{23}-EG_{34}-K_{23}-B_{13}	1	23	37	34	25	46	12863
B_{23}-K_{17}-EG_{34}-K_{17}-B_{23}	1	54	39	34	45	34	15707
B_{24}-K_{25}-EG_{34}-K_{25}-B_{24}	1	54	54	34	48	50	18412
B_{16}-K_{25}-EG_{34}-K_{25}-B_{16}	1	35	54	34	31	49	14561
B_{17}-K_{16}-EG_{34}-K_{16}-B_{17}	1	35	64	34	31	59	15841
B_{14}-K_{19}-EG_2-K_{19}-B_{15}	1[ii)]	30	42	2	27	38	12277

[i)]: determined by ^{1}H NMR.

[ii)]: every molar of PEG-$(NH_2)_2$ is replaced by 1 molar of EDEA.

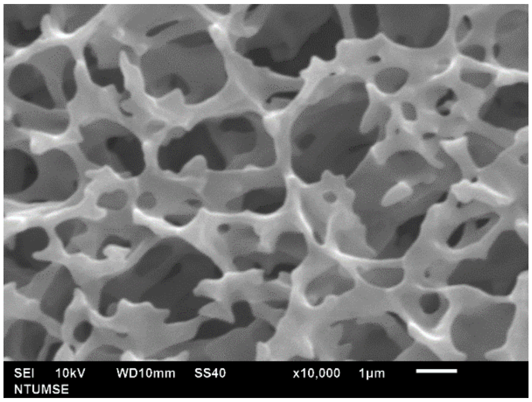

Figure S 6 SEM image of B_{18}-K_{13}-EG_{34}-K_{13}-B_{18}. Interconnected structure with about 2 μm radius is the evidence of trapped water when the hydrogel structure is formed.

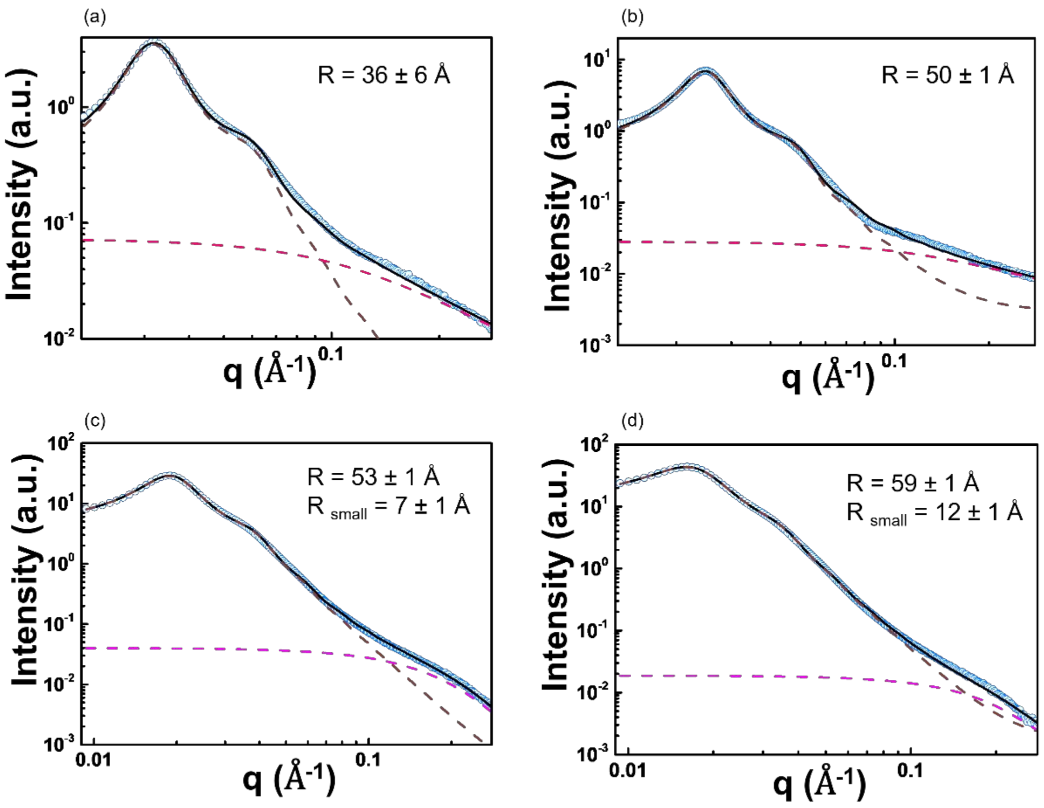

Figure S 7 SAXS profiles of 7.0 wt% (a) B_8-K_{23}-EG_{34}-K_{23}-B_8, (b) B_{10}-K_{20}-EG_{34}-K_{20}-B_{10}, (c) B_{15}-K_{15}-EG_{34}-K_{15}-B_{15}, and (d) B_{18}-K_{13}-EG_{34}-K_{13}-B_{18}. R: Radius. R_{small}: Radius of small aggregates. Noted that the red dotted lines in (a) and (b) are the features of extended polymer chains.

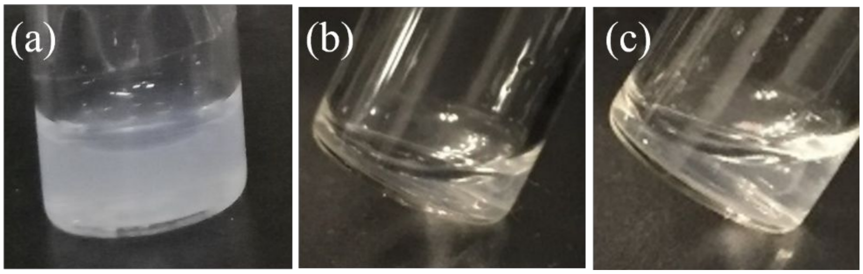

Figure S 8 The appearance of K_y-B_x-EG_{34}-B_x-K_y with different PLL length. (a) K_{15}-B_{15}-EG_{34}-B_{15}-K_{15} (b) K_{27}-B_{15}-EG_{34}-B_{15}-K_{27} (c) K_{44}-B_{16}-EG_{34}-B_{16}-K_{44}.

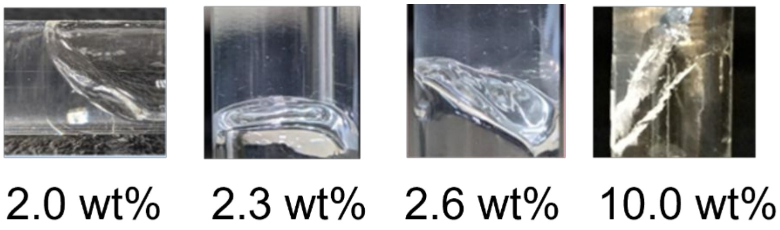

Figure S 9 Transparent appearance of B_{14}-K_{19}-EG_2-K_{19}-B_{14} copolymers solution. The copolymers form hydrogels within the range of concentration from 2.3 wt% to 10.0 wt%.

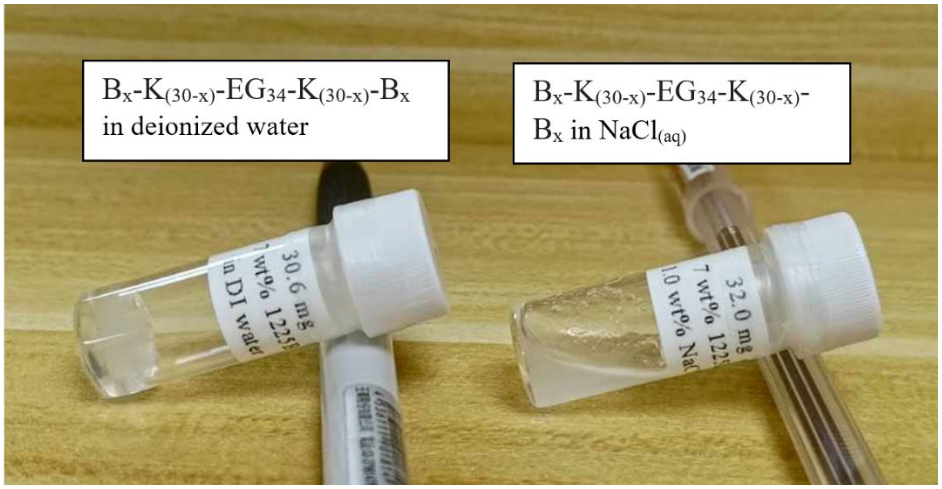

Figure S 10 The salt effect on the self assembly behavior of B-$K_{(30-x)}$-EG_{34}-$K_{(30-x)}$-B_x.
